# Supplementary material for: Disruption of TIGAR-TAK1 alleviates immunopathology in a murine model of sepsis
Source: Nat Commun. 2024 May 21;15:4340. doi: 10.1038/s41467-024-48708-0 (PMC11109194; doi:10.1038/s41467-024-48708-0)

Supplementary information

Disruption of TIGAR-TAK1 interaction alleviates sepsis via suppression of macrophage inflammatory response

Dongdong Wang *et al*.

Corresponding author: bjj@njmu.edu.cn; qichen@njmu.edu.cn.

The PDF file includes:

Supplementary Figures S1 to S13

Supplementary Tables S1

Supplementary uncropped scans of Figures S1 to S13

Supplementary Figures

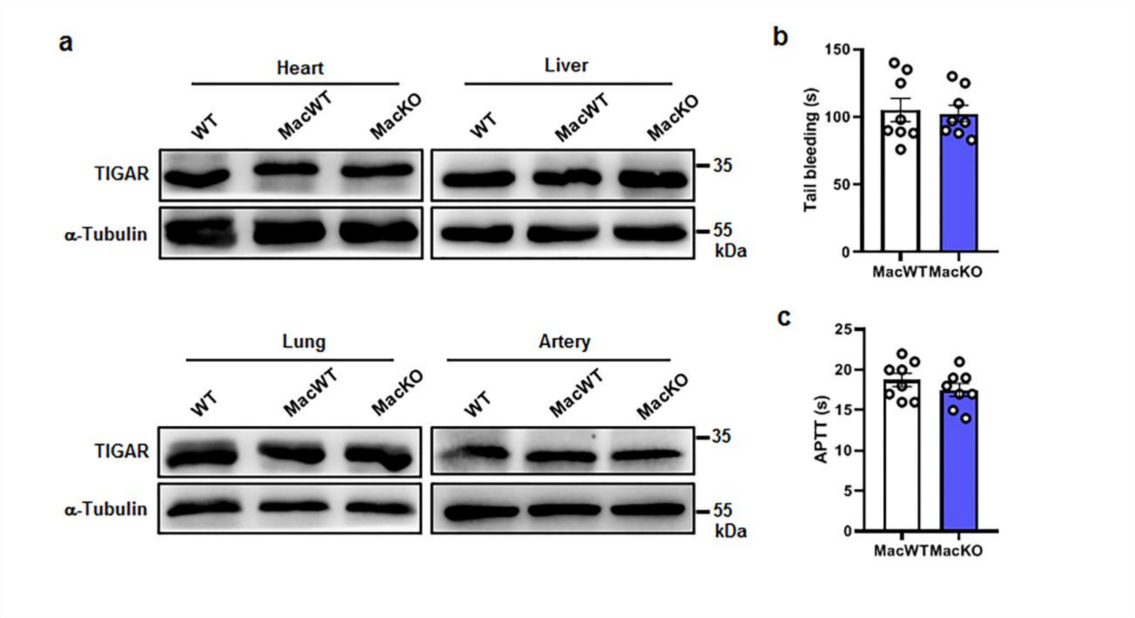

Figure S1. Establishment of myeloid TIGAR specific knockout mice. (**a**) Western blot of TIGAR expression in the indicated tissues from WT, MacWT and MacKO mice. Blot assay was repeated three times independently with similar results. (**b-c**) Tail bleeding time (b) (n = 8) and activated partial thromboplastin time (APTT) (c) (n = 8) of MacWT and MacKO CLP septic mice. Data are expressed as mean ± SEM. **b** and **c**, Two-tailed Student *t* test. Source data are provided as a Source Data file.

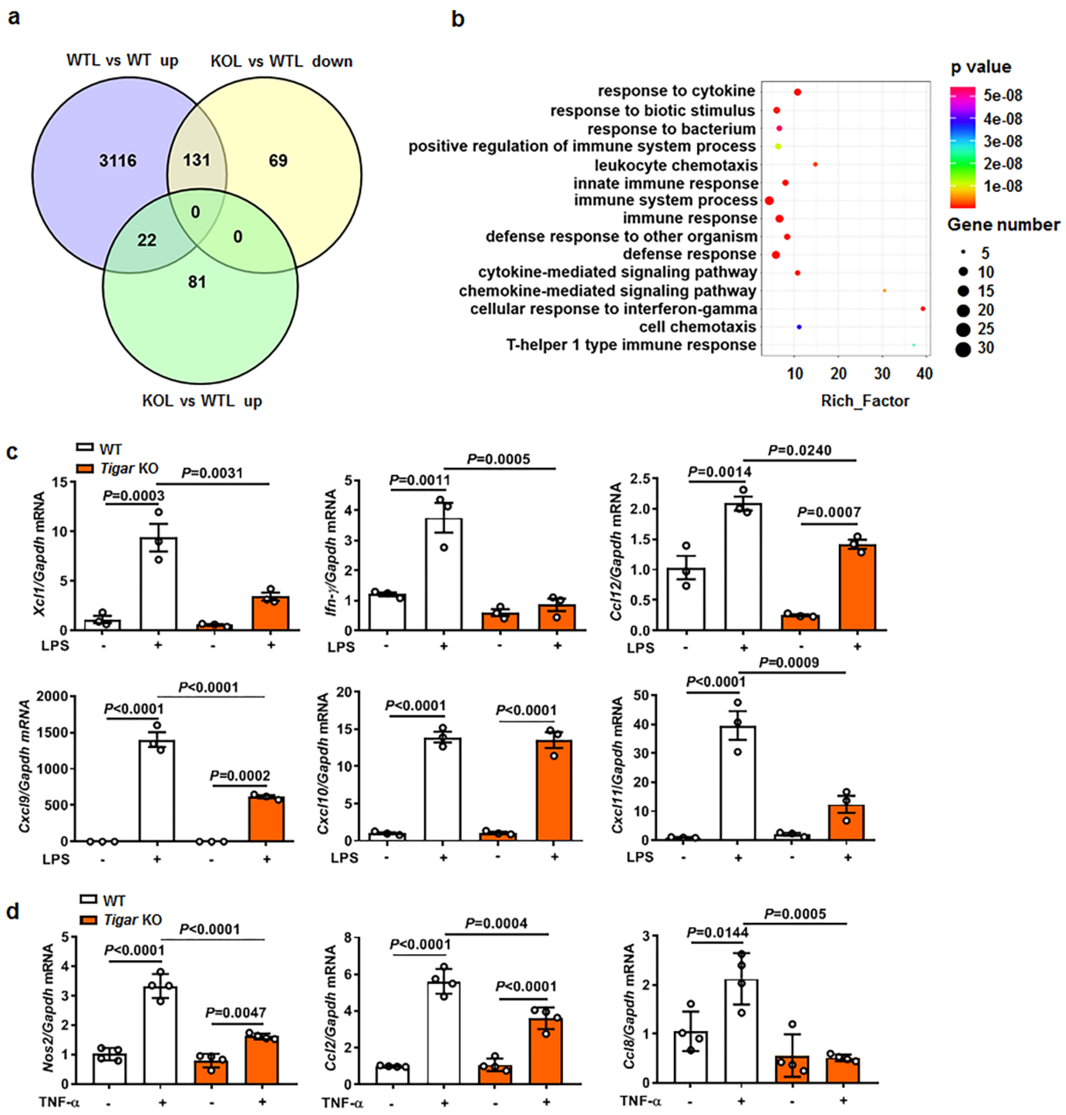

Figure S2. TIGAR activates host pro-inflammatory responses. (a) Venn blot of the different genes screening from the RNA-seq analysis. Different expressed genes (DEGs) defined from the pairwise comparisons were required to satisfy two selection criteria: a fold change > 1.5 and a corresponding adjusted p value < 0.05. BMDMs of WT (W), BMDMs of WT treated by LPS (WTL), BMDMs of *Tigar* KO treated by LPS (KOL). (**b**) Enriched biological processes of the 131 genes that are upregulated in LPS treated WT BMDMs and relatively decreased in LPS treated *Tigar* KO BMDMs. (**c**) mRNA levels of the indicated genes in BMDMs isolated from WT and *TIGAR* KO mice (n =

3). (**d**) After treated by TNF-α (10 ng ml^{-1}) for 1 h, expression of *Nos2*, *Ccl2* and *Ccl8* in cells were measured by RT-qPCR (n = 4). Data are expressed as mean ± SEM. **c** and **d**, One-way ANOVA followed by Bonferroni test. Source data are provided as a Source Data file.

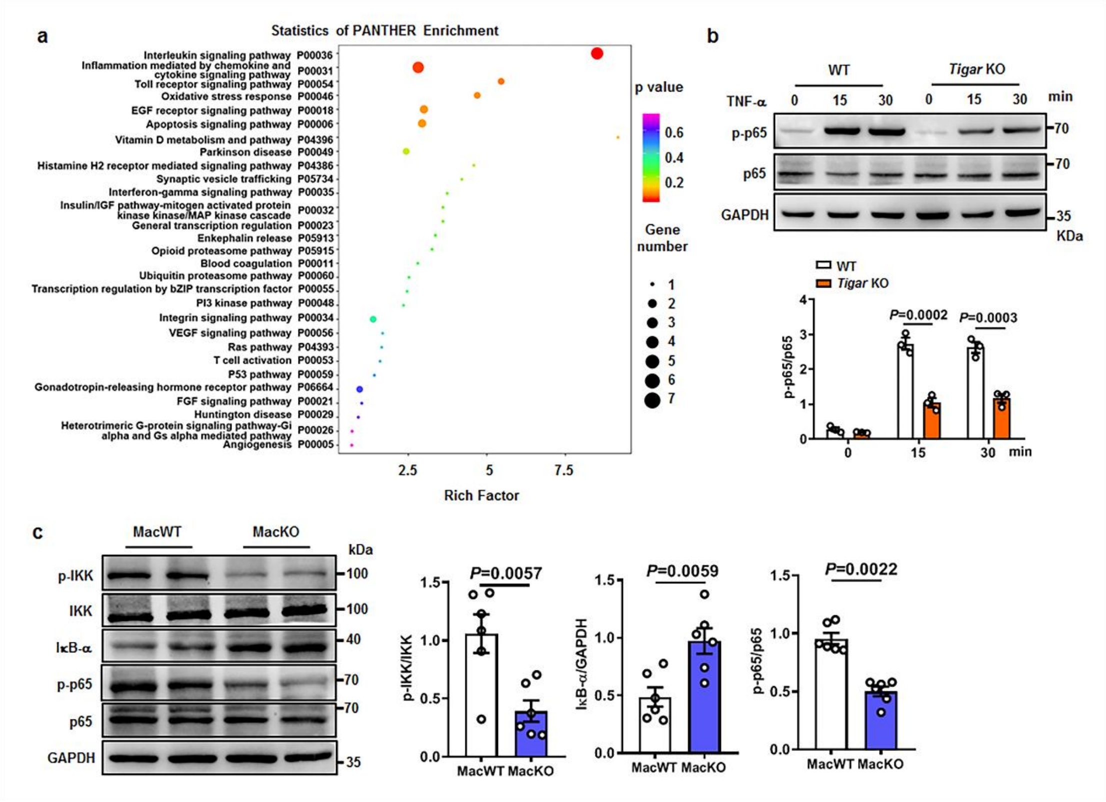

Figure S3. TIGAR deficiency inhibits IKK-NF-κB signaling. (**a**) Panther enrichment analysis of the most significantly altered signaling pathways in LPS-stimulated BMDMs from *Tigar* KO mice. (**b**) Western blot of p-p65 and p65 levels from WT and *Tigar* KO BMDMs stimulated with TNF-α (10 ng ml^{-1}) for indicated times, n = 3 samples. (**c**) Western blot of p-IKK, IKK, IκB-α, p-p65, and p65 in the lung tissues from LPS-induced MacWT and MacKO sepsis mice, n = 6 samples. Data are expressed as mean ± SEM. **b**, Two-way ANOVA followed by Bonferroni test. **c**, Two-tailed Student *t* test. Source data are provided as a Source Data file.

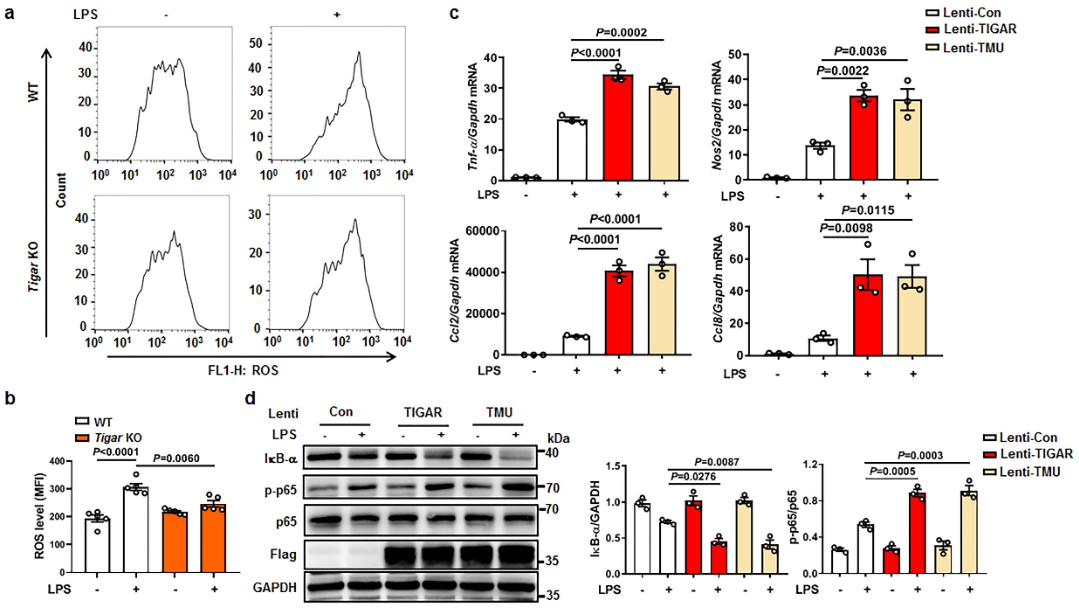

Figure S4. TIGAR promotes inflammation independent of its phosphatase activity. (**a-b**) ROS production in WT and *Tigar* KO BMDMs were measured with the Mean Fluorescence Intensity (MFI) of 20,70-dichlorofluorescein (DCF) by flow cytometry (n = 5). (**c**) RAW264.7 cells were transfected by Lenti-Con, Lenti-TIGAR or Lenti-TMU for 72 h followed by LPS treatment for 12 h. mRNA levels of pro-inflammatory genes were detected in RAW264.7 cells, n = 3 samples. (**d**) RAW264.7 cells were transfected by Lenti-Con, Lenti-TIGAR or Lenti-TMU for 72 h followed by LPS treatment for 30 min. Western blot of IκB-α, p-p65 and p65 in RAW264.7 cells, n = 3 samples. Data are expressed as mean ± SEM. **b-d**, One-way ANOVA followed by Bonferroni test. Source data are provided as a Source Data file.

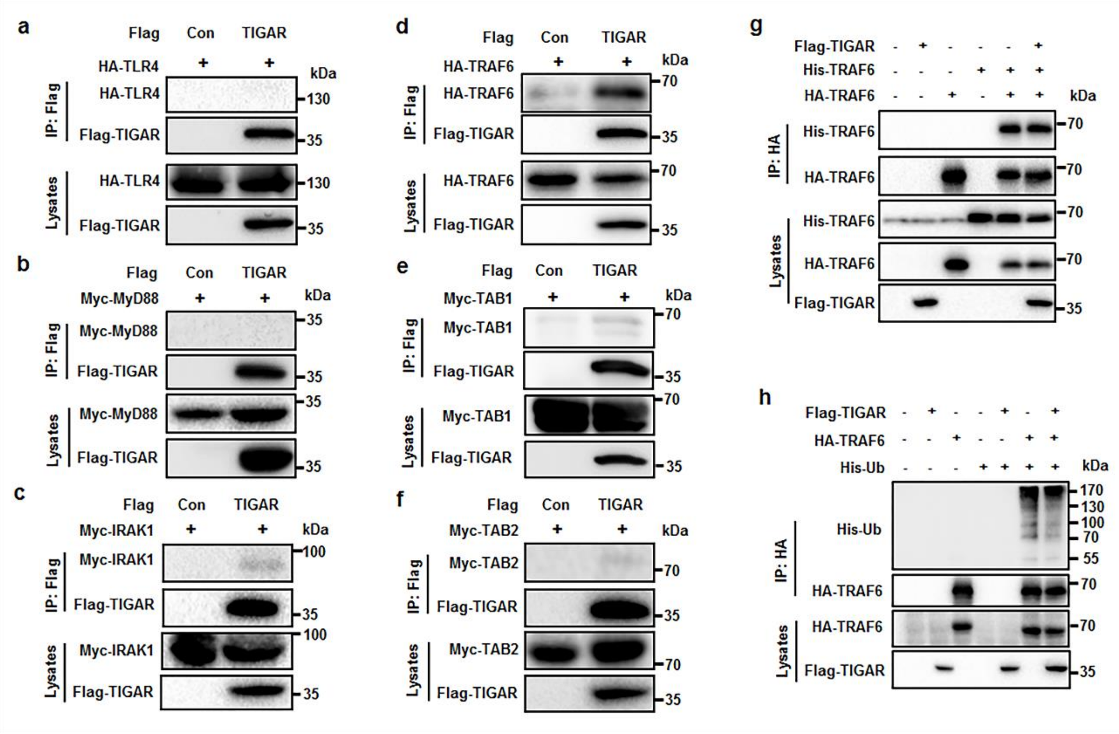

Figure S5. TIGAR binds to TRAF6 but has no effect on TRAF6 dimerization and ubiquitination. (**a-f**) HEK293 cells were transfected by plasmids as indicated. Co-IP and western blot of the interaction of TIGAR to TLR4 (a), MyD88 (b), IRAK1 (c), TRAF6 (d), TAB1 (e) and TAB2 (f). (**g**) Western blot of the effect of TIGAR on the oligomerization of TRAF6 in HEK293 cells. (**h**) Western blot of the effect of TIGAR on the ubiquitination of TRAF6 in HEK293 cells. All blot assays were repeated three times independently with similar results. Source data are provided as a Source Data file.

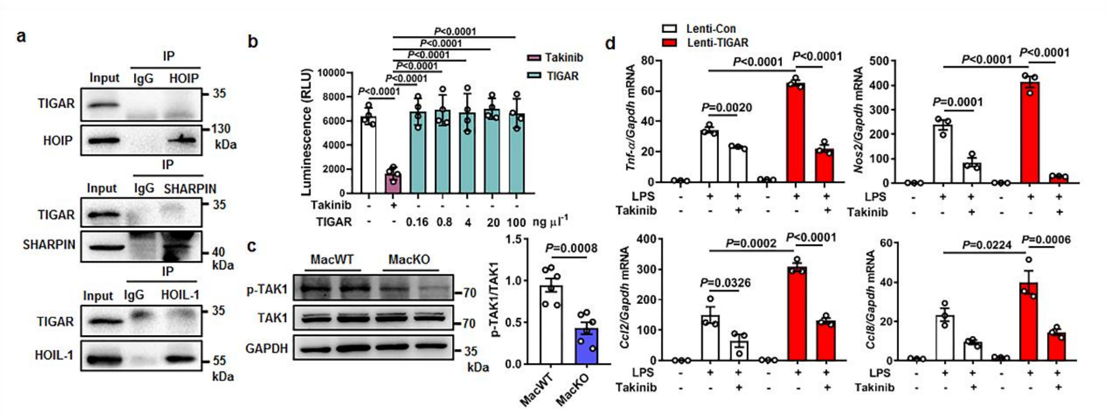

Figure S6. TIGAR deficiency reduces TAK1 phosphorylation. (**a**) Co-IP and western blot of endogenous interaction of TIGAR with HOIP, SHARPIN and HOIL-1 in BMDMs. Blot assay was repeated three times independently with similar results. (**b**) In vitro analysis of the effect of TIGAR and Takinib on TAK1 kinase catalytic activity (n=4). (**c**) Western blot of TAK1 phosphorylation in the lung tissues from LPS-induced MacWT and MacKO sepsis mice, n = 6 samples. (**d**) mRNA levels of pro-inflammatory genes in RAW264.7 cells transfected with Lenti-Con or Lenti-TIGAR followed by LPS treatment with or without Takinib (100 nM) for 12 h (n = 3). Data are expressed as mean ± SEM. **c**, Two-tailed Student *t* test. **b**, **d**, One-way ANOVA followed by Bonferroni test. Source data are provided as a Source Data file.

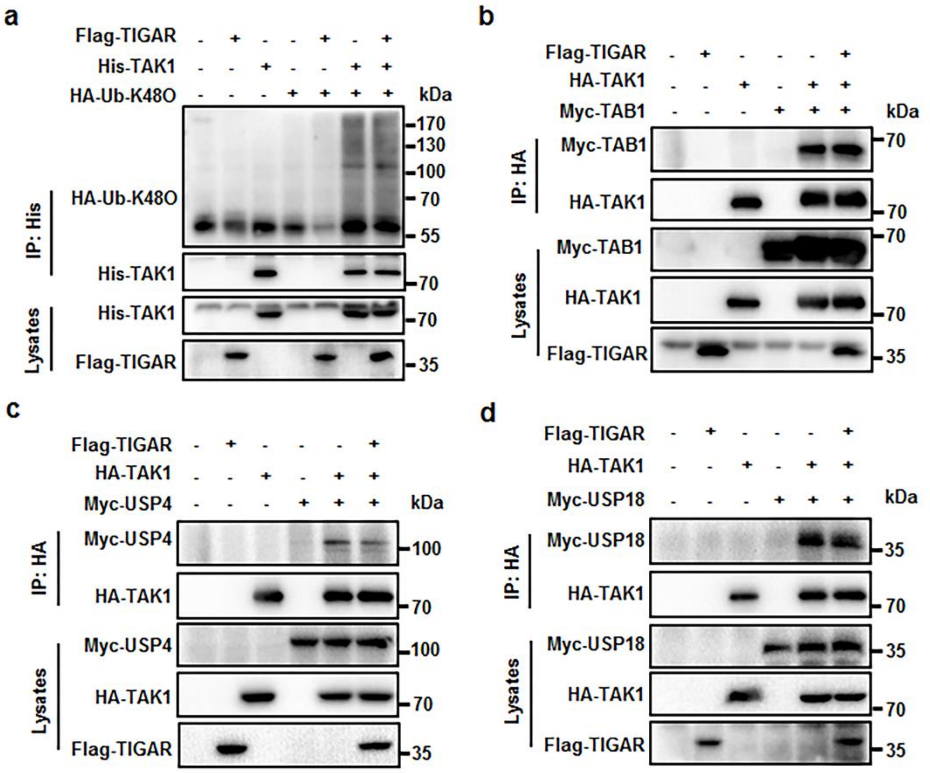

Figure S7. Effect of TIGAR on other regulatory factors of TAK1 ubiquitination. (**a**) HEK293 cells were transfected by His-TAK1, HA-Ub-K48O and Flag-TIGAR plasmids. Co-IP and western blot of the K48-ubiquitination of TAK1. HA-Ub-K48O indicates ubiquitin in which all lysines except K48 were mutated (**b-d**) HEK293 cells were transfected by Flag-TIGAR, HA-TAK1 and indicated plasmids. Co-IP and western blot of the effect of TIGAR on the complex formation between TAK1 and TAB1 (b), USP4 (c) or USP18 (d). All blot assays were repeated three times independently with similar results. Source data are provided as a Source Data file.

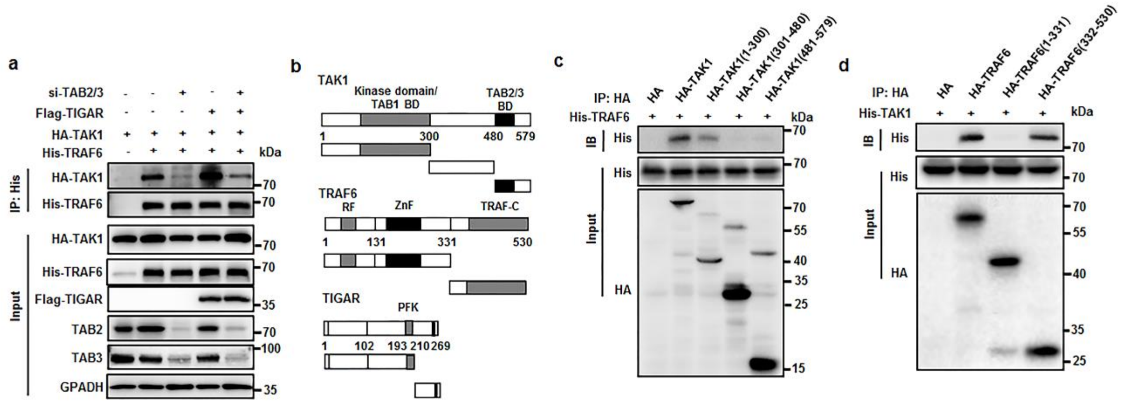

Figure S8. The interaction domains between TRAF6 and TAK1. (**a**) HEK293 cells were transfected by siTAB2/3 or negative control for 48 h followed by transfected by HA-TAK1, His-TRAF6 and Flag-TIGAR plasmids for 24 h. Co-IP and western blot of the complex formation between TAK1 and TRAF6. (**b**) Schematic representation of the full-length and deletion mutants of TAK1, TRAF6 and TIGAR used for determining the interaction domains. (**c**) HEK293 cells were transfected by His-TRAF6 and HA-tagged TAK1 fragments. Co-IP and western blot of the binding regions between TAK1 and TRAF6. (**d**) HEK293 cells were transfected by His-TAK1 and HA-tagged TRAF6 fragments. Co-IP and western blot of the binding regions between TRAF6 and TAK1. All blot assays were repeated three times independently with similar results. Source data are provided as a Source Data file.

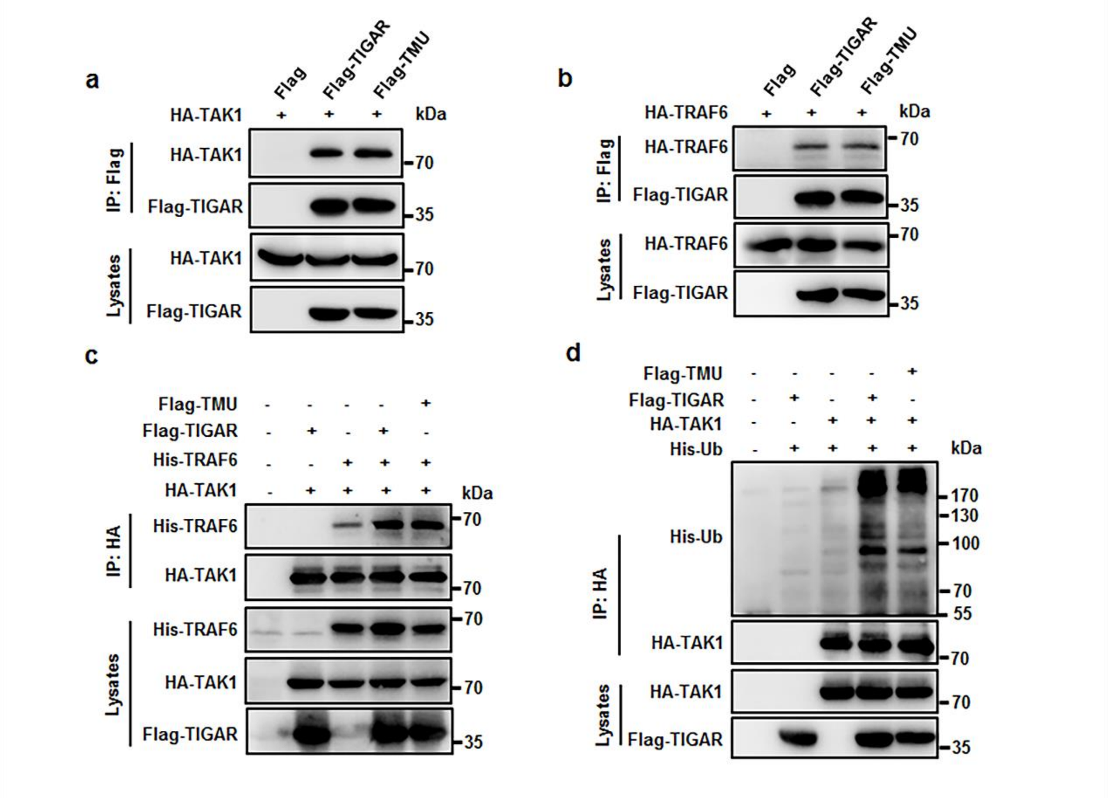

Figure S9. TIGAR promotes TRAF6-TAK1 complex formation and TAK1 ubiquitination independent of its phosphatase activity. (**a**) Co-IP and western blot of the interaction between TAK1 and TIGAR or TMU in HEK293 cells. (**b**) Co-IP and western blot of the interaction between TRAF6 and TIGAR or TMU in HEK293 cells. (**c**) Co-IP and western blot of the effect of TIGAR or TMU on the complex formation between TAK1 and TRAF6 in HEK293 cells. (**d**) Western blot of the effect of TIGAR or TMU on the ubiquitination of TAK1 in HEK293 cells. All blot assays were repeated three times independently with similar results. Source data are provided as a Source Data file.

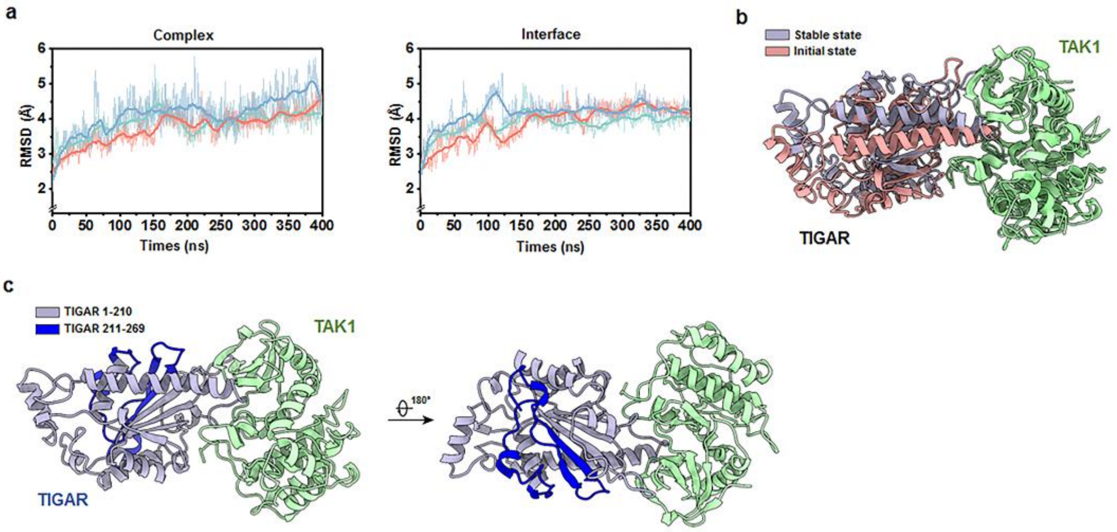

Figure S10. Structure of the TAK1-TIGAR complex obtained from molecular dynamics simulations. (**a**) Root-mean-square deviation (RMSD) fluctuations of TAK1-TIGAR complex system and protein interface based on C-alpha during MD simulations. The raw data of RMSDs are shown in translucent lines, and the fluctuations of RMSD are smoothed by using the Savitzky-Golay method. (**b**) The comparison of the binding modes of TAK1-TIGAR before and after MD refinement indicates the conformational adjustment. (**c**) Schematic diagram of the position of residues 1-210 and 211-269 of TIGAR in the computational TAK-TIGAR complex.

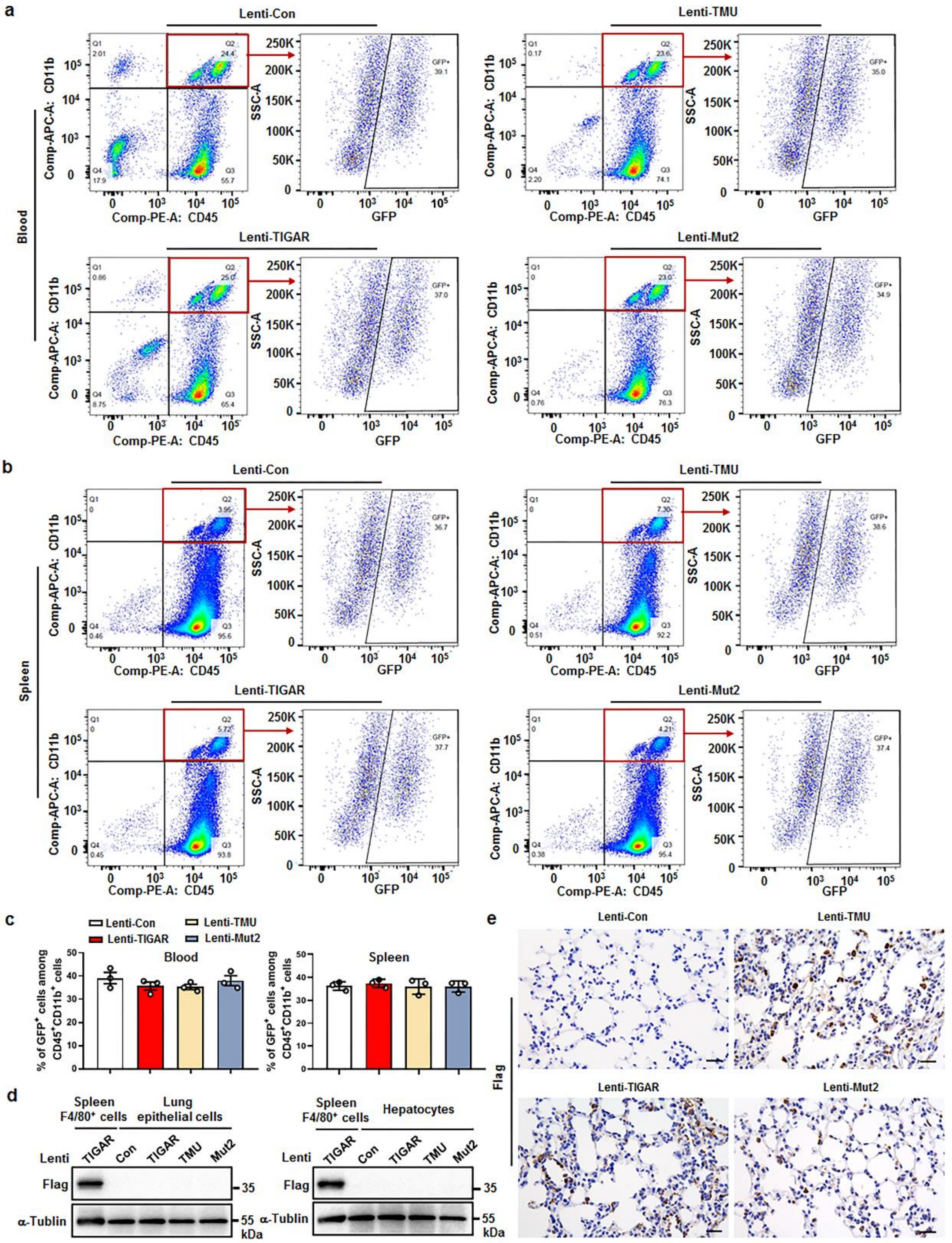

Figure S11. Identification of the transfected TIGAR, TMU and Mut2 in mice. *Tigar* KO mice were infected with myeloid specific CD11b promoter-driven lentivirus (pCDH-CD11b-T2A-copGFP) encoding Flag-TIGAR, Flag-TMU or Flag-Mut2 respectively. After 4 days the CLP sepsis model was generated. (**a** and **b**) Representative flow cytometry plot charts of GFP^+ cells among $CD45^+CD11b^+$ myeloid

cells in blood (a) and spleen (b). (**c**) Quantification of the percentage of GFP^+ cells among $CD45^+CD11b^+$ myeloid cells in blood and spleen (n =3). (**d**) Western blot of Flag expression in spleen $F4/80^+$ cells, lung epithelial cells and hepatocytes. (**e**) Representative Flag staining in lung from the CLP sepsis mice. Scale bars, 20 μm. Data are expressed as mean ± SEM. **c**, One-way ANOVA followed by Bonferroni test. Source data are provided as a Source Data file.

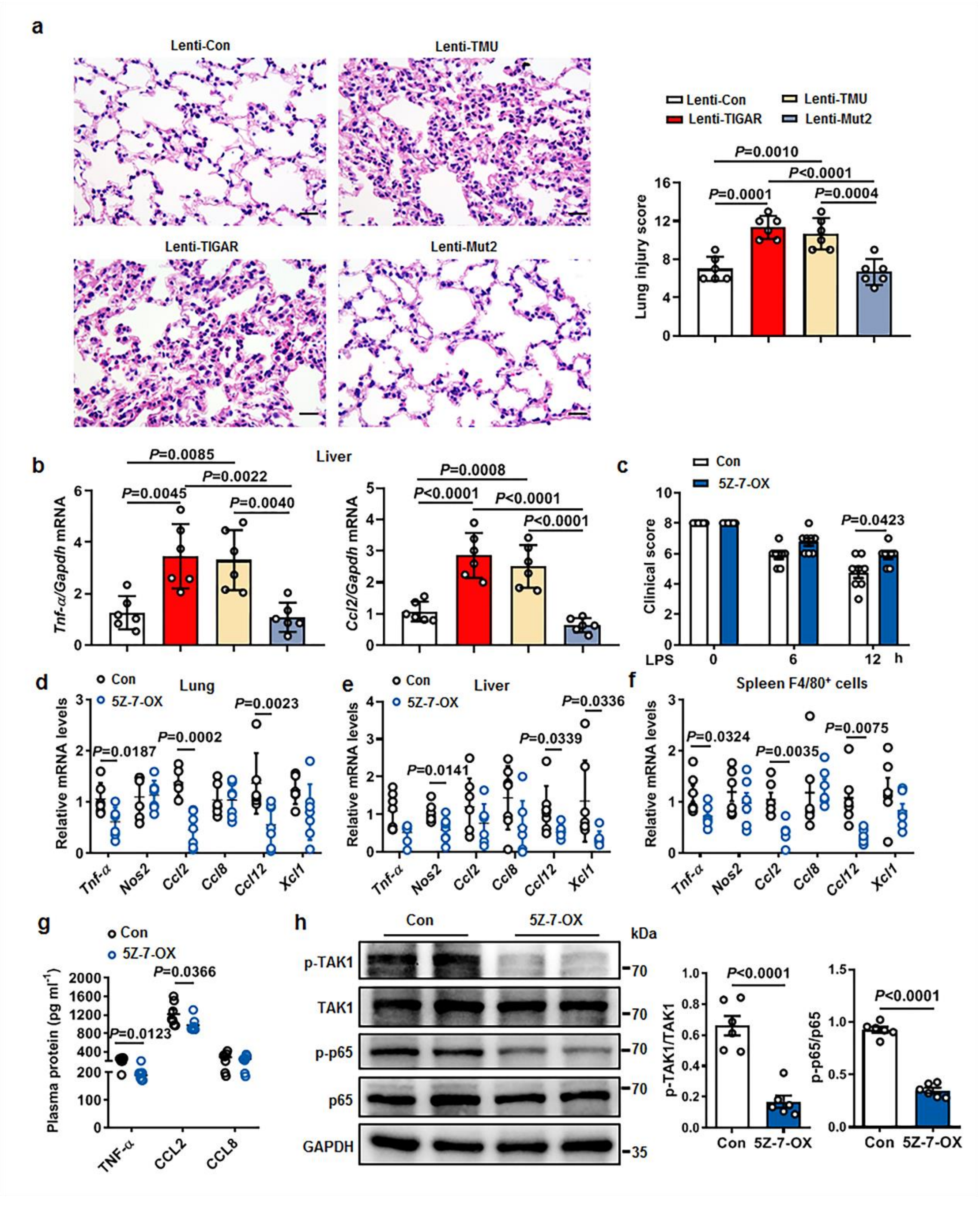

Figure S12. **Inhibiting TIGAR-TAK1 interaction abolishes murine sepsis.** *Tigar* KO mice were infected with myeloid specific CD11b promoter-driven lentivirus encoding Flag-TIGAR, Flag-TMU or Flag-Mut2 respectively. After 4 days the CLP sepsis model was generated. (**a**) Representative H&E staining sections of lung tissues and lung injury score (n = 6). Scale bars, 20 μm. (**b**) mRNA levels of pro-inflammatory genes in liver (n = 6). Male C57BL/6J mice were intraperitoneally injected by 5Z-7-

OX or DMSO (Con). After 1 h, mice were intraperitoneally injected with LPS (10 mg kg^{-1}). The mice were euthanized 12 h later. (**c**) Clinical score of mice was analyzed (n = 8). (**d-f**) mRNA levels of pro-inflammatory genes in lung (d) (Con, n = 6, 5Z-7-OX, n = 7), liver (e) (n = 7), and spleen F4/80$^+$ cells (f) (n = 6). (**g**) Plasma concentrations of TNF-α, CCL2, and CCL8 in Con (n = 7) and 5Z-7-OX (n = 8) treated mice. (**h**) Western blot of p-TAK1, TAK1, p-p65 and p65 levels in the lung tissues from Con and 5Z-7-OX treated mice, n = 6 samples. Data are expressed as mean ± SEM. **a**, **b**, One-way ANOVA followed by Bonferroni test. **c, h**, Two-tailed Student *t* test. **d, e, f** and **g**, Two-tailed Student *t* test or two-tailed Mann-Whitney *U* test. Source data are provided as a Source Data file.

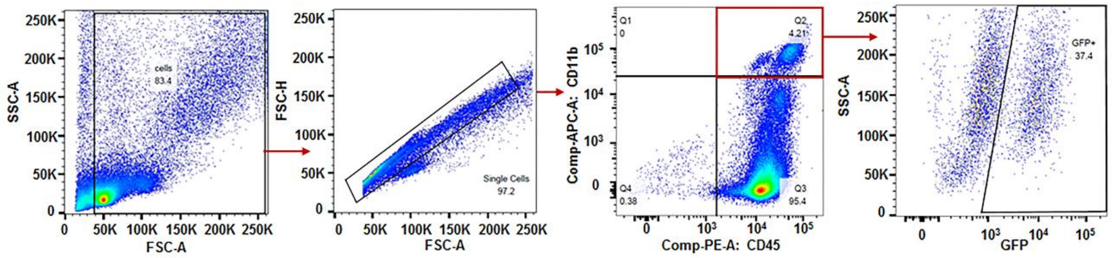

Figure S13. Flow cytometry gating strategy for GFP$^+$ cells among CD45$^+$CD11b$^+$ myeloid cells.

Supplementary table

Table S1. Primer pairs used for RT-qPCR.

Gene	Forward primer (5`-3`)	Reverse primer (5`-3`)
Tnf-α	ACGGCATGGATCTCAAAGAC	AGATAGCAAATCGGCTGACG
Nos2	GTTCTCAGCCCAACAATACAAGA	GTGGACGGGTCGATGTCAC
Ccl2	TTAAAAACCTGGATCGGAAC	GCATTAGCTTCAGATTTACGGGT
Ccl8	TCTACGCAGTGCTTCTTTGCC	AAGGGGGATCTTCAGCTTTAGTA
Ccl12	ATTTCCACACTTCTATGCCTCCT	ATCCAGTATGGTCCTGAAGATCA
Xcl1	TTTGTCACCAAACGAGGACTAAA	CCAGTCAGGGTTATCGCTGTG
Gapdh	AGGTCGGTGTGAACGGATTTG	TGTAGACCATGTAGTTGAGGTCA

Figure S1

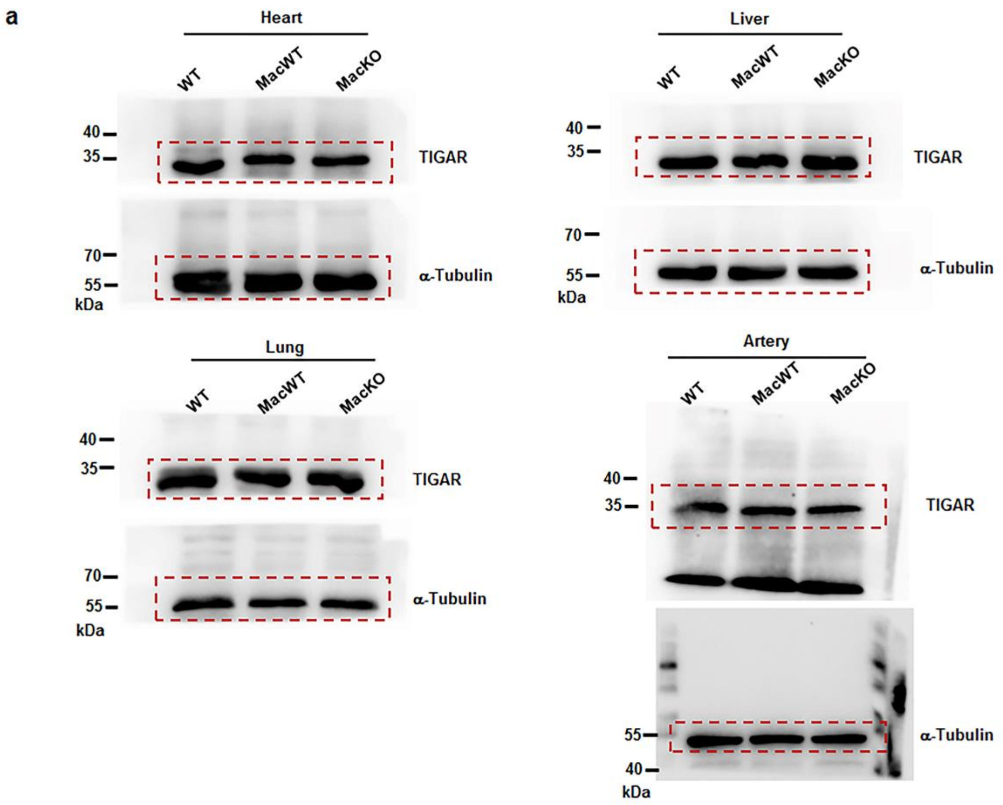

Figure S3

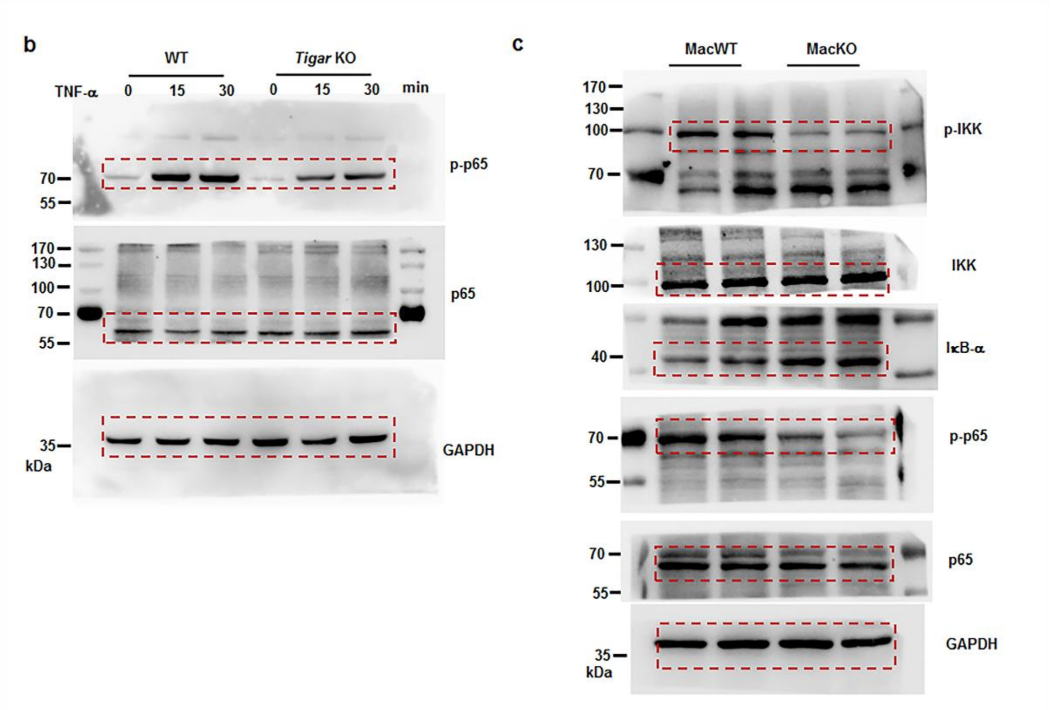

Figure S4

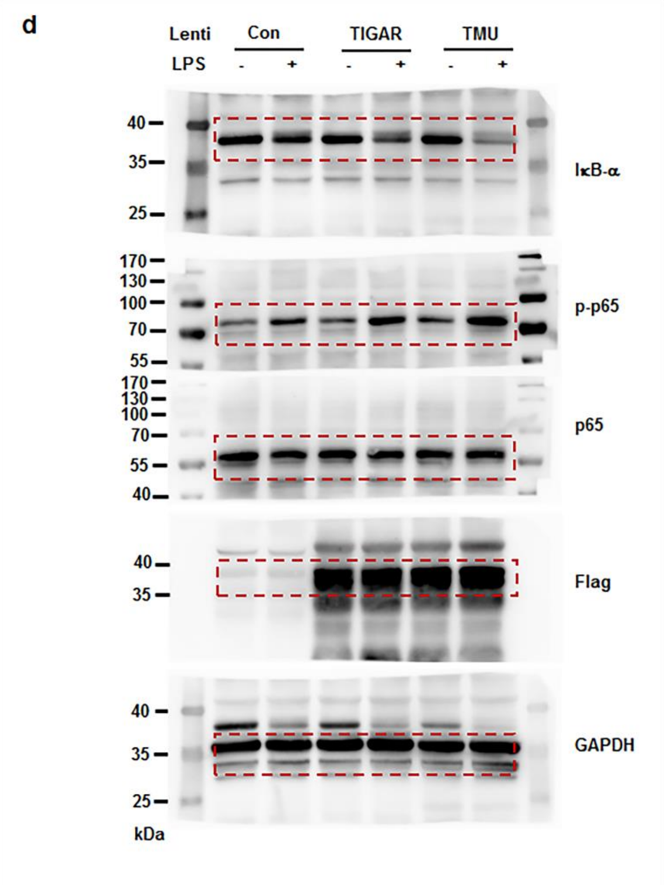

Figure S5

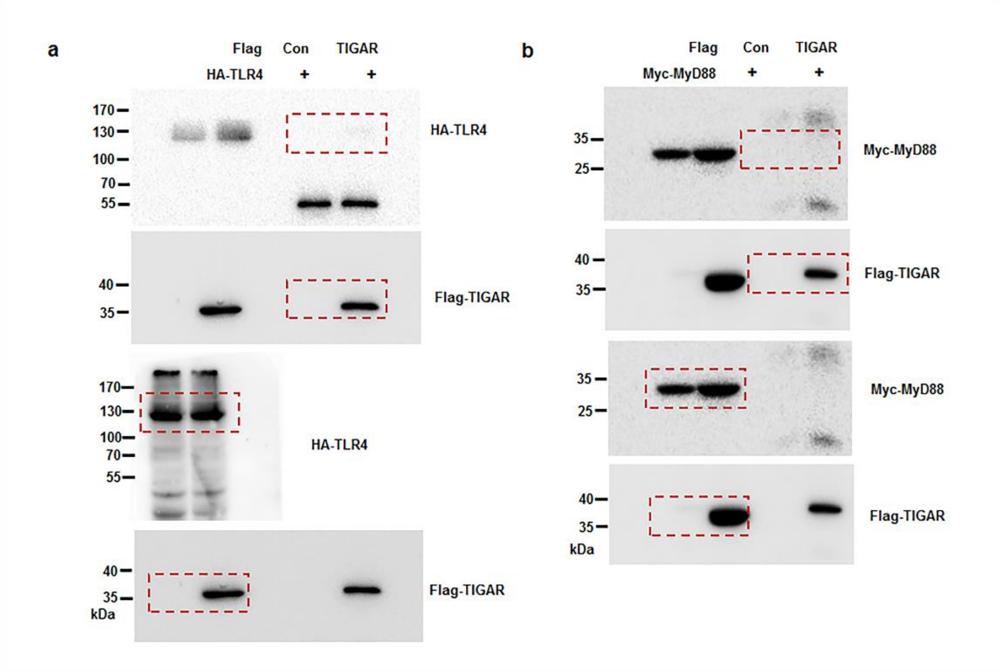

Figure S5

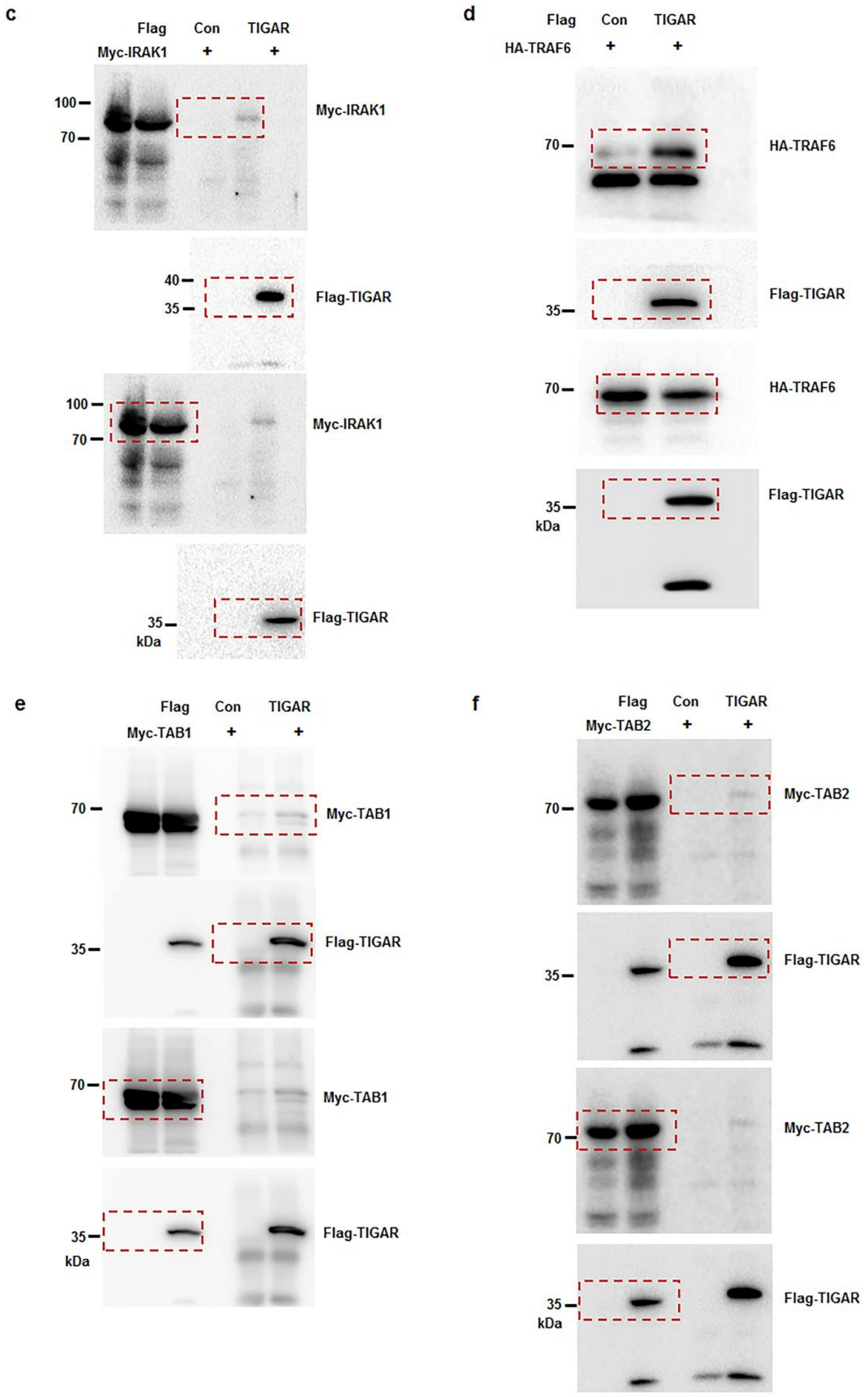
c
Flag
Con
TIGAR
Myc-IRAK1
+
+
100
70
Myc-IRAK1
40
35
Flag-TIGAR
100
70
Myc-IRAK1
35
kDa
Flag-TIGAR
d
Flag
Con
TIGAR
HA-TRAF6
+
+
70
HA-TRAF6
35
Flag-TIGAR
70
HA-TRAF6
35
kDa
Flag-TIGAR
e
Flag
Con
TIGAR
Myc-TAB1
+
+
70
Myc-TAB1
35
Flag-TIGAR
70
Myc-TAB1
35
kDa
Flag-TIGAR
f
Flag
Con
TIGAR
Myc-TAB2
+
+
70
Myc-TAB2
35
Flag-TIGAR
70
Myc-TAB2
35
kDa
Flag-TIGAR

Figure S5

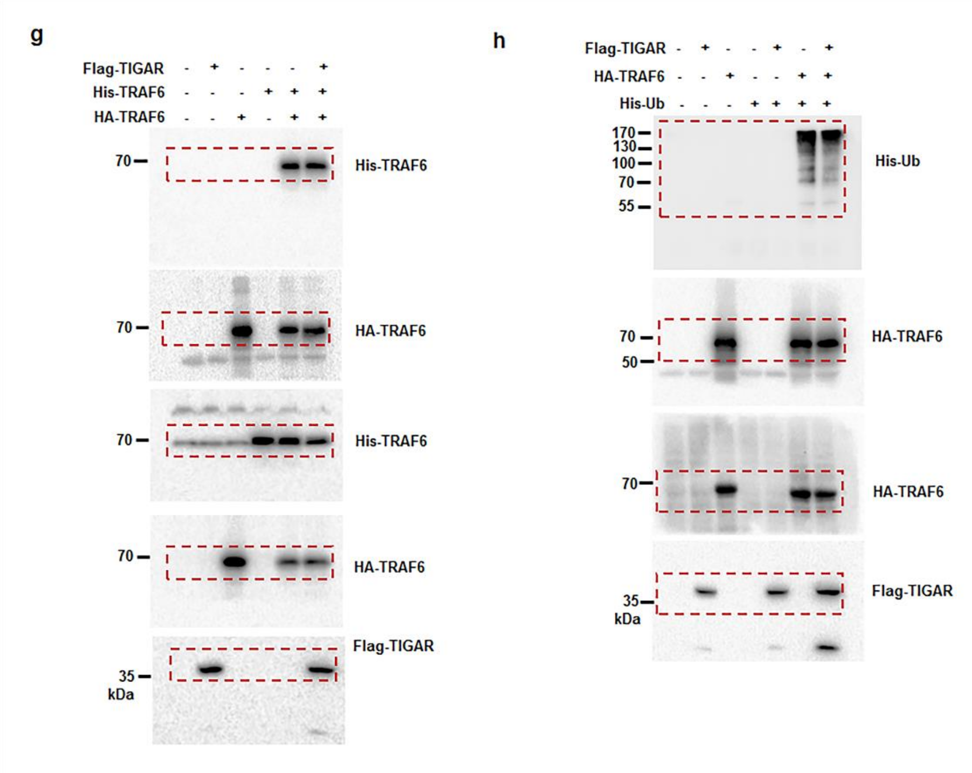

Figure S6

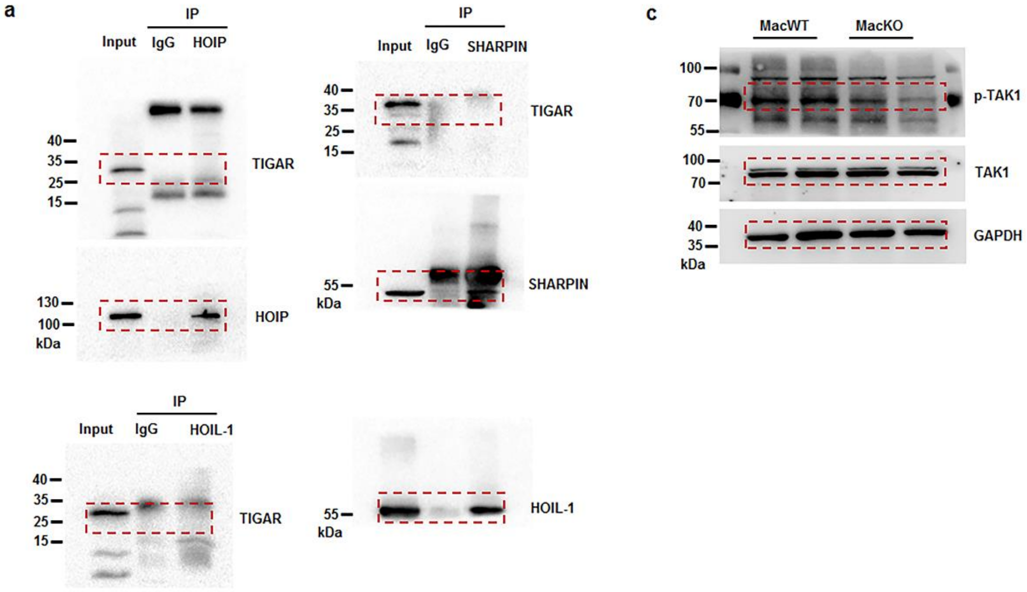

Figure S7

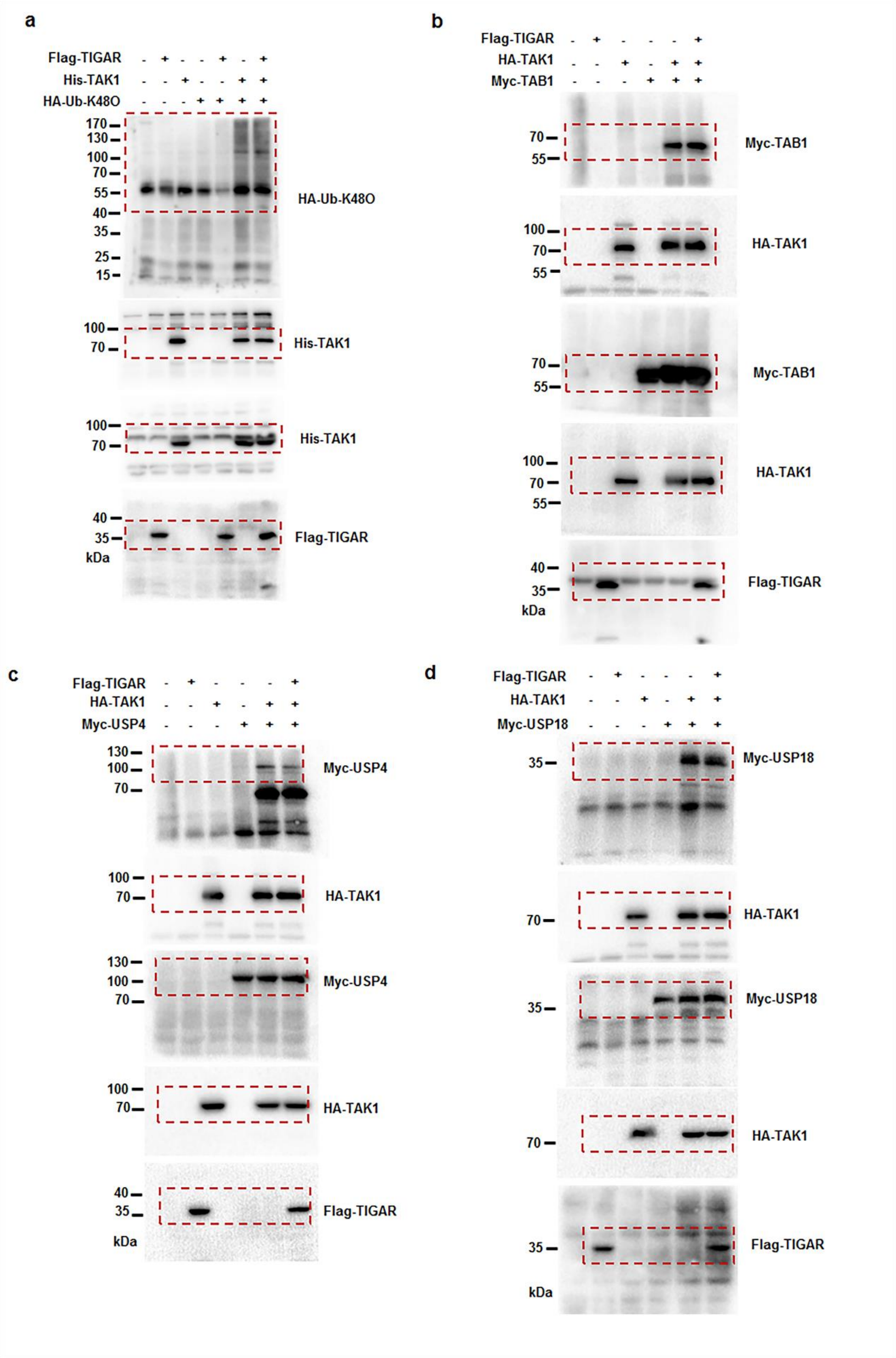

a
Flag-TIGAR - + - - + - +
His-TAK1 - - + - - + +
HA-Ub-K48O - - - + + + +
170
130
100
70
55
40
35
25
15
HA-Ub-K48O
100
70
His-TAK1
100
70
His-TAK1
40
35
kDa
Flag-TIGAR
b
Flag-TIGAR - + - - - +
HA-TAK1 - - + - + +
Myc-TAB1 - - - + + +
70
55
Myc-TAB1
100
70
55
HA-TAK1
70
55
Myc-TAB1
100
70
55
HA-TAK1
40
35
kDa
Flag-TIGAR
c
Flag-TIGAR - + - - - +
HA-TAK1 - - + - + +
Myc-USP4 - - - + + +
130
100
70
Myc-USP4
100
70
HA-TAK1
130
100
70
Myc-USP4
100
70
HA-TAK1
40
35
kDa
Flag-TIGAR
d
Flag-TIGAR - + - - - +
HA-TAK1 - - + - + +
Myc-USP18 - - - + + +
35
Myc-USP18
70
HA-TAK1
35
Myc-USP18
70
HA-TAK1
35
kDa
Flag-TIGAR

Figure S8

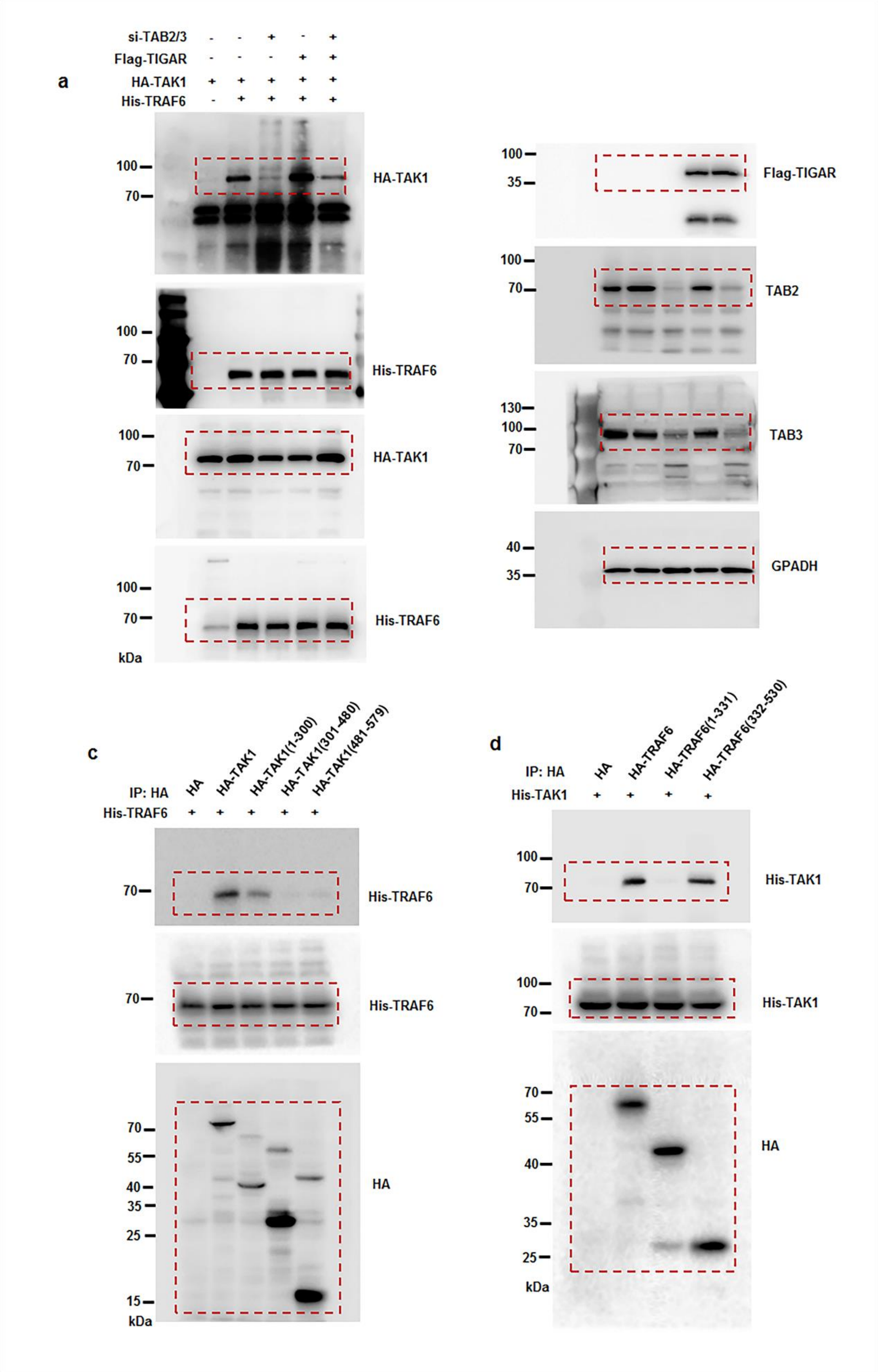
a
si-TAB2/3
Flag-TIGAR
HA-TAK1
His-TRAF6
HA-TAK1
His-TRAF6
HA-TAK1
His-TRAF6
kDa
Flag-TIGAR
TAB2
TAB3
GPADH
c
IP: HA
His-TRAF6
HA
HA-TAK1
HA-TAK1(1-300)
HA-TAK1(301-480)
HA-TAK1(481-579)
His-TRAF6
His-TRAF6
HA
kDa
d
IP: HA
His-TAK1
HA
HA-TRAF6
HA-TRAF6(1-331)
HA-TRAF6(332-530)
His-TAK1
His-TAK1
HA
kDa

Figure S9

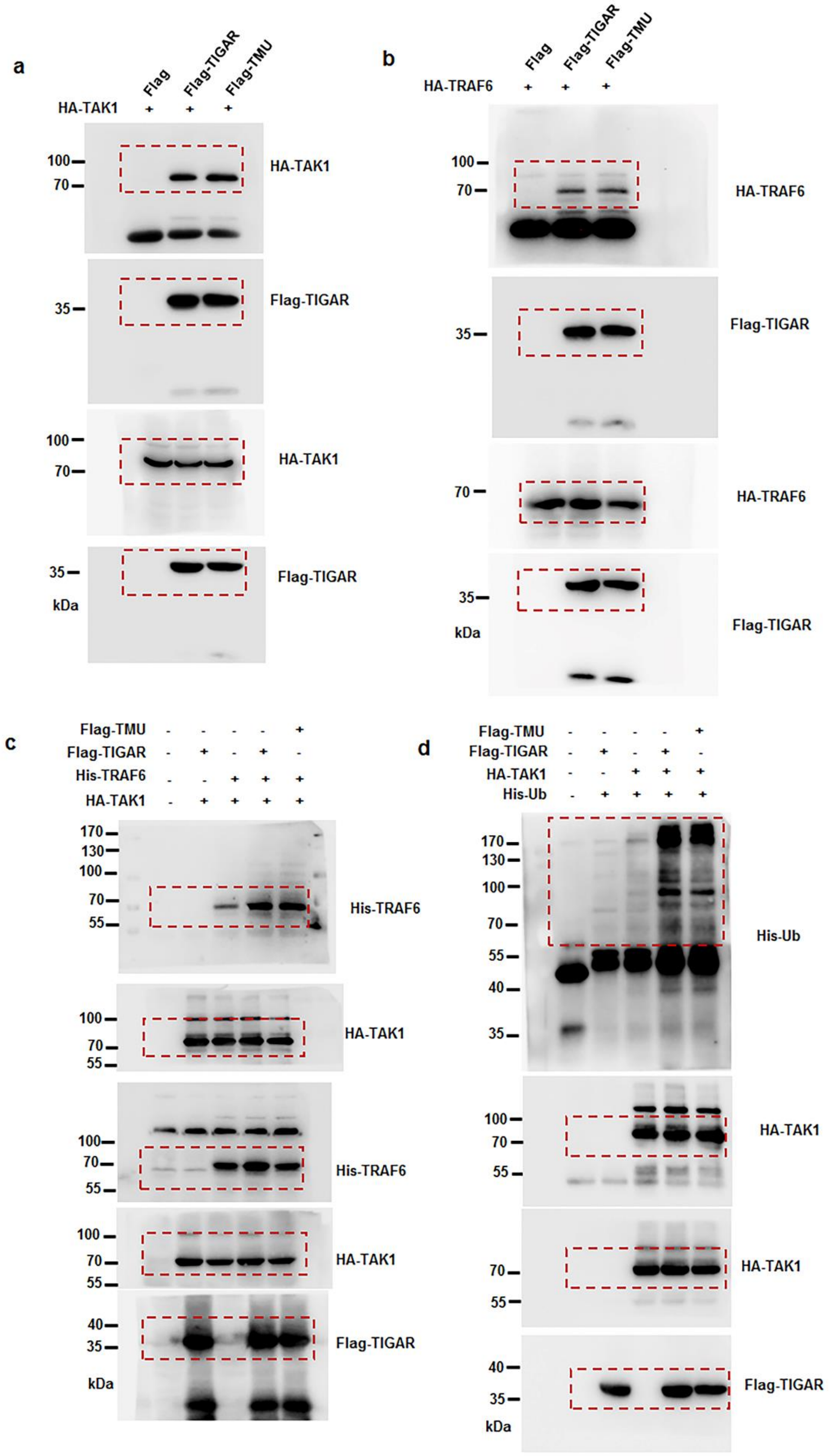
a
Flag
Flag-TIGAR
Flag-TMU
HA-TAK1 + + +
100
70
HA-TAK1
35
Flag-TIGAR
100
70
HA-TAK1
35
kDa
Flag-TIGAR
b
Flag
Flag-TIGAR
Flag-TMU
HA-TRAF6 + + +
100
70
HA-TRAF6
35
Flag-TIGAR
70
HA-TRAF6
35
kDa
Flag-TIGAR
c
Flag-TMU - - - - +
Flag-TIGAR - + - + -
His-TRAF6 - - + + +
HA-TAK1 - + + + +
170
130
100
70
55
His-TRAF6
100
70
55
HA-TAK1
100
70
55
His-TRAF6
100
70
55
HA-TAK1
40
35
kDa
Flag-TIGAR
d
Flag-TMU - - - - +
Flag-TIGAR - + - + -
HA-TAK1 - - + + +
His-Ub - + + + +
170
130
100
70
55
40
35
His-Ub
100
70
55
HA-TAK1
70
55
HA-TAK1
40
35
kDa
Flag-TIGAR

Figure S11

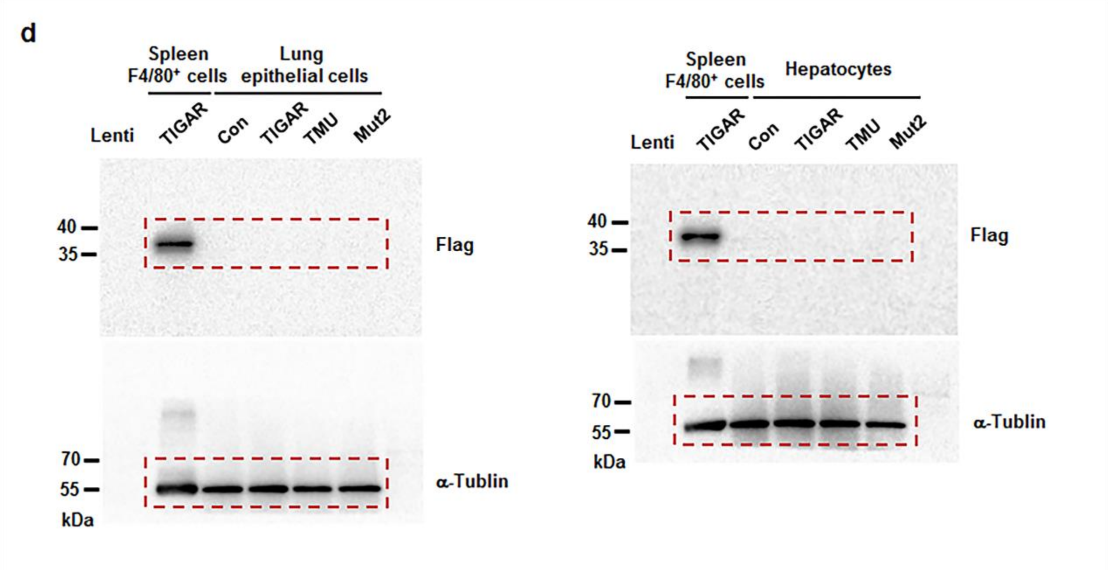

Figure S12

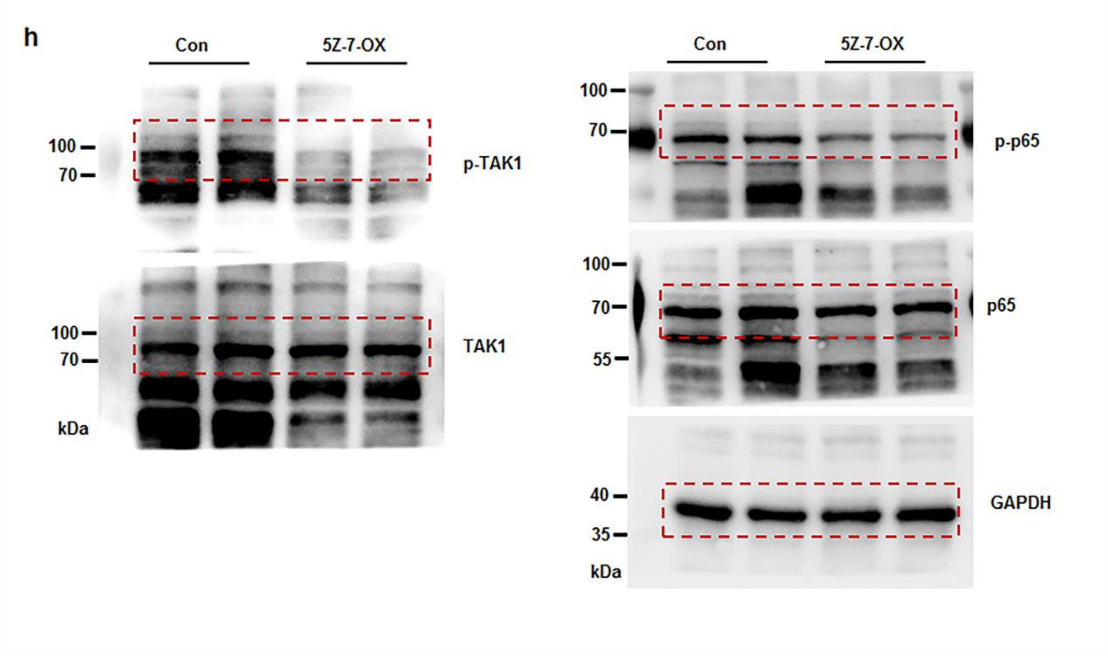

Supplement: Supplementary file 1 — Supplementary Information [file 41467_2024_48708_MOESM1_ESM.pdf]
