# Supplementary material for: Disruption of TIGAR-TAK1 alleviates immunopathology in a murine model of sepsis
Source: Nat Commun. 2024 May 21;15:4340. doi: 10.1038/s41467-024-48708-0 (PMC11109194; doi:10.1038/s41467-024-48708-0)
Supplement: Supplementary file 3 — Reporting Summary [file 41467_2024_48708_MOESM3_ESM.pdf]

nature portfolio

Corresponding author(s): Qi Chen, Jingjing Ben

Last updated by author(s): Apr 16, 2024

Reporting Summary

Nature Portfolio wishes to improve the reproducibility of the work that we publish. This form provides structure for consistency and transparency in reporting. For further information on Nature Portfolio policies, see our Editorial Policies and the Editorial Policy Checklist.

Statistics

For all statistical analyses, confirm that the following items are present in the figure legend, table legend, main text, or Methods section.

n/a	Confirmed	
☐	☒	The exact sample size (*n*) for each experimental group/condition, given as a discrete number and unit of measurement
☐	☒	A statement on whether measurements were taken from distinct samples or whether the same sample was measured repeatedly
☐	☒	The statistical test(s) used AND whether they are one- or two-sided<br>*Only common tests should be described solely by name; describe more complex techniques in the Methods section.*
☒	☐	A description of all covariates tested
☐	☒	A description of any assumptions or corrections, such as tests of normality and adjustment for multiple comparisons
☐	☒	A full description of the statistical parameters including central tendency (e.g. means) or other basic estimates (e.g. regression coefficient) AND variation (e.g. standard deviation) or associated estimates of uncertainty (e.g. confidence intervals)
☐	☒	For null hypothesis testing, the test statistic (e.g. *F*, *t*, *r*) with confidence intervals, effect sizes, degrees of freedom and *P* value noted<br>*Give P values as exact values whenever suitable.*
☒	☐	For Bayesian analysis, information on the choice of priors and Markov chain Monte Carlo settings
☒	☐	For hierarchical and complex designs, identification of the appropriate level for tests and full reporting of outcomes
☒	☐	Estimates of effect sizes (e.g. Cohen's *d*, Pearson's *r*), indicating how they were calculated

Our web collection on statistics for biologists contains articles on many of the points above.

Software and code

Policy information about availability of computer code

Data collection	BD FACSuite software v9.1 was used for cytofluorimetric data. Zeiss ZEN 2012 was used for image collection.
Data analysis	GraphPad Prism (v8); FlowJo (v10); ImageJ (c1.48); ImagePro Plus (v6)

For manuscripts utilizing custom algorithms or software that are central to the research but not yet described in published literature, software must be made available to editors and reviewers. We strongly encourage code deposition in a community repository (e.g. GitHub). See the Nature Portfolio guidelines for submitting code & software for further information.

Data

Policy information about availability of data

All manuscripts must include a data availability statement. This statement should provide the following information, where applicable:

- Accession codes, unique identifiers, or web links for publicly available datasets
- A description of any restrictions on data availability
- For clinical datasets or third party data, please ensure that the statement adheres to our policy

The raw bulk RNA sequencing data have been deposited in the gene expression omnibus under accession no. GSE202446 [https://www.ncbi.nlm.nih.gov/geo/query/acc.cgi?acc=GSE202446]. All data supporting the findings of this study are available within the main manuscript and the supplementary files.

Research involving human participants, their data, or biological material

Policy information about studies with human participants or human data. See also policy information about sex, gender (identity/presentation), and sexual orientation and race, ethnicity and racism.

Reporting on sex and gender	N/A
Reporting on race, ethnicity, or other socially relevant groupings	N/A
Population characteristics	N/A
Recruitment	N/A
Ethics oversight	N/A

Note that full information on the approval of the study protocol must also be provided in the manuscript.

Field-specific reporting

Please select the one below that is the best fit for your research. If you are not sure, read the appropriate sections before making your selection.

[x] Life sciences   [ ] Behavioural & social sciences   [ ] Ecological, evolutionary & environmental sciences

For a reference copy of the document with all sections, see nature.com/documents/nr-reporting-summary-flat.pdf

Life sciences study design

All studies must disclose on these points even when the disclosure is negative.

Sample size	We did not use statistical method to predetermine sample size. The sample size was based on the previous literature that a minimum of N = 3 biological replicates with sufficient reproducibility in cell experiments and a minimum of N = 6 biological replicates with sufficient reproducibility in animal experiments (PMID: 31902237; PMID: 30405100). The number of the independent experiments for cell and biological experiments was indicated in each figure legend.
Data exclusions	No data were excluded from the analyses.
Replication	Most experiments were validated with repeat experiments at least 3 times and the results were consistent between experiments.
Randomization	Treatment group in mouse experiments were mixed within the same cage and groups were assigned randomly. For in vitro experiments, all cells in each experiment were randomly from mice in each group.
Blinding	Animal treatments were performed by technicians who were not involved in sample measurement. All in vitro experiments were not blind because students have their own projects, and it is impossible for others to replace them to treat cells.

Reporting for specific materials, systems and methods

We require information from authors about some types of materials, experimental systems and methods used in many studies. Here, indicate whether each material, system or method listed is relevant to your study. If you are not sure if a list item applies to your research, read the appropriate section before selecting a response.

Materials & experimental systems

n/a	Involved in the study	
[ ]	[x]	Antibodies
[ ]	[x]	Eukaryotic cell lines
[x]	[ ]	Palaeontology and archaeology
[ ]	[x]	Animals and other organisms
[x]	[ ]	Clinical data
[x]	[ ]	Dual use research of concern
[x]	[ ]	Plants

Methods

n/a	Involved in the study	
[x]	[ ]	ChIP-seq
[ ]	[x]	Flow cytometry
[x]	[ ]	MRI-based neuroimaging

Antibodies

Antibodies used	The following primary antibodies were used for western blot (WB). They are listed as antigen first, followed by catalog number, clone

name, dilution ratio, and supplier.
1） TIGAR (sc-166290, E-2, 1:500, Santa Cruz)
2） TAK1 (sc-166562, H-5, 1:500, Santa Cruz)
3） phospho-TAK1 (MA5-15073, NA, 1:1000, Thermo Fisher Scientific)
4） phospho-IKKα/β (2697, NA, 1:1000, Cell Signaling Technology)
5） IKKα/β (sc-7607, NA, 1:500, Santa Cruz)
6） IκBα (4814, NA,1:1000, Cell Signaling)
7） phospho-p65 (3033, NA,1:1000, Cell Signaling Technology)
8） p65 (8242, 1:1000, NA, Cell Signaling Technology)
9） HOIP (RNF31) (A303-560A, NA, 1:500, Bethyl)
10） SHARPIN (ab197853, NA, 1:1000, Abcam)
11） HOIL-1 (RBCK1) (sc-365523, E-2, 1:200, Santa Cruz)
12） Flag (F1804, M2, 1:1000, Sigma-Aldrich)
13） HA (11867423001, 3F10, 1:1000, Roche)
14） HA (26183, 2-2.2.14, 1:5000, Thermo Fisher Scientific)
15） Ub (MAB1510, 042691GS, 1:1000, Millipore)
16） K63-Ub (5621, 1:1000, NA, Cell Signaling Technology)
17） His (66005-1-Ig, NA, 1:1000, Protein Tech)
18） Myc (2278, NA, 1:1000, Cell Signaling Technology)
19） α-Tubulin (11224-1-AP, NA, 1:1000, Protein Tech)
20） GAPDH (KC-5G4, NA, 1:3000, Kangchen Tech)
21） β-actin (sc-47778, C4, 1:1000, Santa Cruz)
22） Lamin B1 (66095-1-Ig, 1:1000, Protein Tech)
The following primary antibodies were used for Flow cytometry antibodies. They are listed as antigen first, followed by catalog number, clone name, dilution ratio, and supplier.
1） APC anti-CD11b (553312, M1/70, 1:100, BD Pharmingen)
2） PE anti-CD45 (553081, 30-F11, 1:100, BD Pharmingen)
The following primary antibodies were used for IHC antibodies. They are listed as antigen first, followed by catalog number, clone name, dilution ratio, and supplier.
1） Flag antibody (rabbit, 14793, NA, 1:100, Cell Signaling Technology)
The following primary antibodies were used for Co-IP antibodies. They are listed as antigen first, followed by catalog number, clone name, dilution ratio, and supplier.
1） anti-TAK1 (5206, NA, 1:200, Cell Signaling Technology)
2） anti-TRAF6 (PA5-29622, NA, 1:200, Thermo Fisher Scientific)
3） anti-Flag (F1804, M2, 1:500, Sigma-Aldrich)
4） anti-HA (26183, 2-2.2.14, 1:500, Thermo Fisher Scientific)
5） anti-His (MA1-21315, HIS.H8, 1:500,Thermo Fisher Scientific)
The following primary antibodies were used for immunofluorescence antibodies. They are listed as antigen first, followed by catalog number, clone name, dilution ratio, and supplier.
1） anti-TIGAR (rabbit, NA, ab189164, 1:100, Abcam)
2） anti-p65 (rabbit, 8242, D14E12, 1:100, Cell Signaling)
3） anti-TAK1 (sc-166562, H-5, 1:100, Santa Cruz)
4） Donkey anti-Rabbit IgG (H+L) Highly Cross-Adsorbed Secondary Antibody, Alexa Fluor™ 488 (A-21206, NA, 1:1000, Thermo Fisher Scientific)
5） Donkey anti-Mouse IgG (H+L) Highly Cross-Adsorbed Secondary Antibody, Alexa Fluor™ 555 (A-31570, NA, 1:1000, Thermo Fisher Scientific)

Validation

All antibodies were commercially available, and validated by manufacturers and/or citations. Manufacturer websites containing their validation data and/or citations, are listed below:
WB antibodies:
1） TIGAR (sc-166290, Santa Cruz)
https://www.scbt.com/zh/p/tigar-antibody-e-2
2） TAK1 (sc-166562, Santa Cruz)
https://www.scbt.com/zh/p/tak1-antibody-h-5
3） phospho-TAK1 (MA5-15073, Thermo Fisher Scientific)
https://www.thermofisher.cn/cn/zh/antibody/product/Phospho-TAK1-Thr184-Thr187- Antibody-clone-K-846-3-Monoclonal/MA5-15073
4） phospho-IKKα/β (2697, Cell Signaling Technology)
https://www.cellsignal.cn/products/primary-antibodies/phospho-ikka-b-ser176-180-16a6-rabbit-mab/2697
5） IKKα/β (sc-7607, Santa Cruz)
https://www.scbt.com/zh/p/ikkalpha-beta-antibody-h-470
6） IκBα (4814, Cell Signaling)
https://www.cellsignal.cn/products/primary-antibodies/ikba-l35a5-mouse-mab-amino- terminal-antigen/4814
7） phospho-p65 (3033, Cell Signaling Technology)

https://www.cellsignal.cn/products/primary-antibodies/phospho-nf-kb-p65-ser536-93h1- rabbit-mab/3033
8）p65 (8242, Cell Signaling Technology)
https://www.cellsignal.cn/products/primary-antibodies/nf-kb-p65-d14e12-xp-174-rabbit- mab/8242
9）HOIP (RNF31) (A303-560A, Bethyl)
https://www.biomol.com/products/antibodies/primary-antibodies/general/anti-rnf31-a303-560a-t?number=A303-560A
10）SHARPIN (ab197853, Abcam)
https://www.abcam.cn/products/primary-antibodies/sharpin-antibody-ab197853.html
11）HOIL-1 (RBCK1) (sc-365523, Santa Cruz)
https://www.scbt.com/zh/p/rbck1-antibody-e-2
12）Flag (F1804, Sigma-Aldrich)
https://www.sigmaaldrich.cn/CN/zh/search/f1804?focus=products&page=1&perpage=30&sort=relevance&term=F1804&type=product
13）HA (11867423001, Roche)
https://www.sigmaaldrich.cn/CN/zh/search/11867423001?focus=products&page=1&perpage=30&sort=relevance&term=11867423001&type=product
14）HA (26183, Thermo Fisher Scientific)
https://www.sigmaaldrich.cn/CN/zh/substance/monoclonalantihatagantibodyproducedinmouse1234598765
15）Ub (MAB1510, Millipore)
https://www.sigmaaldrich.cn/CN/zh/search/mab1510?focus=products&page=1&perpage=30&sort=relevance&term=MAB1510&type=product
16）K63-Ub (5621, Cell Signaling Technology)
https://www.cellsignal.cn/products/primary-antibodies/k63-linkage-specific-polyubiquitin- d7a11-rabbit-mab/5621
17）His (66005-1-Ig, Protein Tech)
https://www.ptgcn.com/products/His-Tag-Antibody-66005-1-Ig.htm
18）Myc (2278, Cell Signaling Technology)
https://www.cellsignal.cn/products/primary-antibodies/myc-tag-71d10-rabbit-mab/2278
19）α-Tubulin (11224-1-AP, Protein Tech)
https://www.ptgcn.com/products/TUBA1B-Antibody-11224-1-AP.htm
20）GAPDH (KC-5G4, Kangchen Tech)
http://jinpanbio.com/archives/133755
21）β-actin (sc-47778, Santa Cruz)
https://www.scbt.com/zh/p/beta-actin-antibody-c4
22）Lamin B1 (66095-1-Ig, Protein Tech)
https://www.ptgcn.com/products/LMNB1-Antibody-66095-1-Ig.htm
Flow cytometry antibodies:
1）APC anti-CD11b (553312, BD Pharmingen)
https://www.bdbiosciences.com/zh-cn/products/reagents/flow-cytometry-reagents/research- reagents/single-color-antibodies-ruo/apc-rat-anti-cd11b.553312
2）PE anti-CD45 (553081, BD Pharmingen)
https://www.bdbiosciences.com/zh-cn/products/reagents/flow-cytometry-reagents/research- reagents/single-color-antibodies-ruo/pe-rat-anti-mouse-cd45.553081
IHC antibodies:
1）Flag antibody (rabbit, 14793, Cell Signaling Technology)
https://www.cellsignal.cn/products/primary-antibodies/dykddddk-tag-d6w5b-rabbit-mab-binds-to-same-epitope-as-sigma-aldrich-anti-flag-m2-antibody/14793

Co-IP antibodies:
1）anti-TAK1 (5206, Cell Signaling Technology)
https://www.cellsignal.cn/products/primary-antibodies/tak1-d94d7-rabbit-mab/5206
2) anti-TRAF6 (PA5-29622, Thermo Fisher Scientific)
https://www.thermofisher.cn/cn/zh/antibody/product/TRAF6-Antibody-Polyclonal/PA5-29622
3）anti-Flag (F1804, Sigma-Aldrich)
https://www.sigmaaldrich.cn/CN/zh/search/f1804?focus=products&page=1&perpage=30&sort=relevance&term=F1804&type=product
4）anti-HA (26183, Thermo Fisher Scientific)
https://www.thermofisher.cn/cn/zh/antibody/product/HA-Tag-Antibody-clone-2-2-2-14- Monoclonal/26183
5）anti-His (MA1-21315, Thermo Fisher Scientific)
https://www.thermofisher.cn/cn/zh/antibody/product/6x-His-Tag-Antibody-clone-HIS-H8- Monoclonal/MA1-21315

Immunofluorescence antibodies:
1）anti-TIGAR (rabbit, NA, ab189164, 1:100, Abcam)
https://www.abcam.cn/products/primary-antibodies/tigar-antibody-ab189164.html
2）anti-p65 (rabbit, 8242, D14E12, 1:100, Cell Signaling)
https://www.cellsignal.cn/products/primary-antibodies/nf-kb-p65-d14e12-xp-174-rabbit-mab/8242
3）anti-TAK1 (sc-166562, H-5, 1:100, Santa Cruz)
https://www.scbt.com/zh/p/tak1-antibody-h-5
4）Donkey anti-Rabbit IgG (H+L) Highly Cross-Adsorbed Secondary Antibody, Alexa Fluor™ 488 (A-21206, NA, 1:1000, Thermo Fisher Scientific)

https://www.thermofisher.cn/cn/zh/antibody/product/Donkey-anti-Rabbit-IgG-H-L-Highly-Cross-Adsorbed-Secondary-Antibody-Polyclonal/A-21206
5）Donkey anti-Mouse IgG (H+L) Highly Cross-Adsorbed Secondary Antibody, Alexa Fluor™ 555 (A-31570, NA, 1:1000, Thermo Fisher Scientific)

Eukaryotic cell lines

Policy information about cell lines and Sex and Gender in Research

Cell line source(s)	HEK293T and RAW264.7 were obtained from the American Type Culture Collection (ATCC).
Authentication	Cell lines were not authenticated by ourselves.
Mycoplasma contamination	All cell lines used in this study tested negative for mycoplasma before their use.
Commonly misidentified lines (See ICLAC register)	No commonly misidentified cell lines were used in this study.

Animals and other research organisms

Policy information about studies involving animals; ARRIVE guidelines recommended for reporting animal research, and Sex and Gender in Research

Laboratory animals	All animal protocols were approved by the Institutional Animal Care and Use Committee of Nanjing Medical University. Mice were housed in temperature-(20-24 °C) and humidity- (30%-70%) controlled,12:12 h light-cycled conventional animal quarters with free access to water and food. Details are described in the methods section. Tigar KO mice were kindly provided by Dr. Yaoyu Chen (Nanjing Medical University, China), and Tigar flox/flox mice were provided by Dr. Zhenghong Qin (Soochow University, China). To generate the myeloid Tigar-deficient mice, Tigarflox/flox mice were crossed with Lyz2-Cre mice (B6.129P2-Lyz2tm1(cre)Ifo/J, Lyz2-CreKI/KI). 8-week-old male mice were included in this study.
Wild animals	The study did not involve wild animals.
Reporting on sex	Male mice were used in this study to avoid possible influence of estrogen on survival and inflammation in septic mice.
Field-collected samples	The study did not involve field-collected samples.
Ethics oversight	Institutional Animal Care and Use Committee of Nanjing Medical University.

Note that full information on the approval of the study protocol must also be provided in the manuscript.

Plants

Seed stocks	N/A
Novel plant genotypes	N/A
Authentication	N/A

Flow Cytometry

Plots

Confirm that:

- [x] The axis labels state the marker and fluorochrome used (e.g. CD4-FITC).
- [x] The axis scales are clearly visible. Include numbers along axes only for bottom left plot of group (a 'group' is an analysis of identical markers).
- [x] All plots are contour plots with outliers or pseudocolor plots.
- [x] A numerical value for number of cells or percentage (with statistics) is provided.

Methodology

Sample preparation	WT and Tigar KO BMDMs were treated with LPS (100 ng/ml) for 8 h. Then, 10 uM DCFH-DA was added and cells were incubated for 30 min. The cells were washed three times with serum-free cell culture solution. Fluorescence intensity was determined with a flow cytometer. Murine spleens were excised, minced and filtered in PBS. Cell suspension was filtered through a 200 µm cell strainer and centrifugated at 1500 rpm for 10 min at 4 °C. Whole blood was collected from the facial vein and red blood cells were lysed using RBC lysis buffer. Cells were resuspended in PBS supplemented with 1% FBS and stained with indicated fluorescent isotope conjugated antibodies for 30 min at room temperature in the dark.
Instrument	FACS Aria II, FACS Verse
Software	All events were acquired using BD FACSDiva and FACSuite software and data were analyzed with FlowJo v10 software.
Cell population abundance	Cells were not sorted.
Gating strategy	Cells were gated by FSC-A x SSC-A to exclude debris and then by FSC-A x FSC-H to exclude cell doublets. For regular flow, myeloid cells were identified as CD45+CD11b+ cells from blood and spleen of mice.

☒ Tick this box to confirm that a figure exemplifying the gating strategy is provided in the Supplementary Information.
