# Supplementary figures and images for: Disruption of TIGAR-TAK1 alleviates immunopathology in a murine model of sepsis
Source: Nat Commun. 2024 May 21;15:4340. doi: 10.1038/s41467-024-48708-0 (PMC11109194; doi:10.1038/s41467-024-48708-0)

**Fig 1**

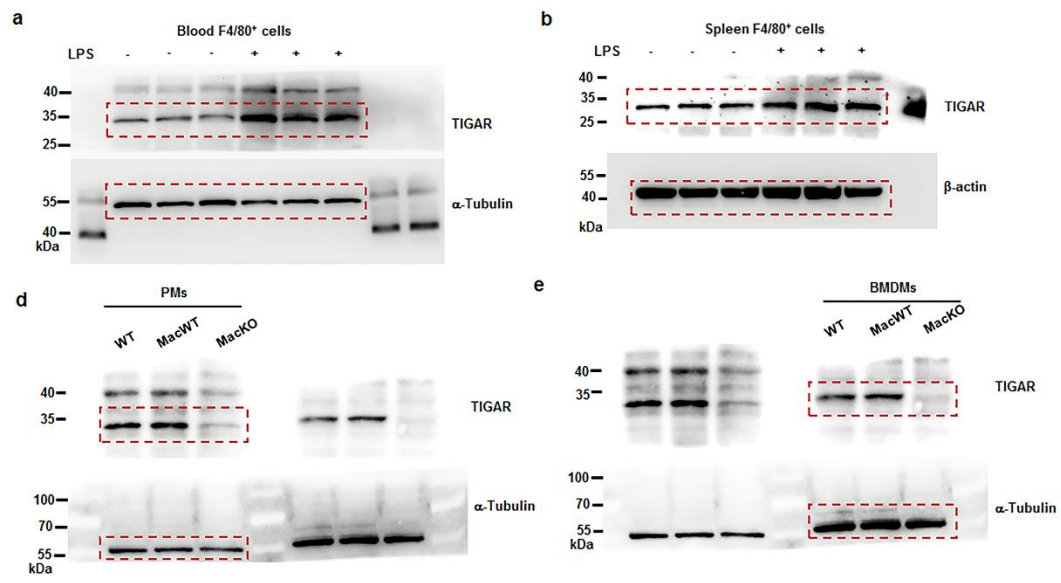

**Fig 2**

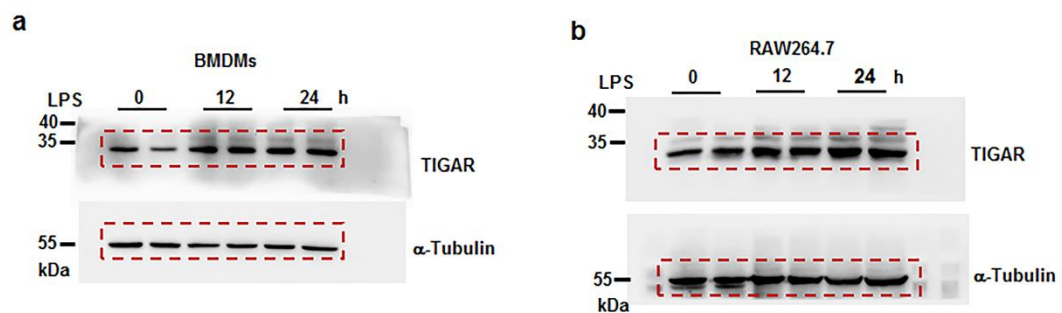

**Fig 3**

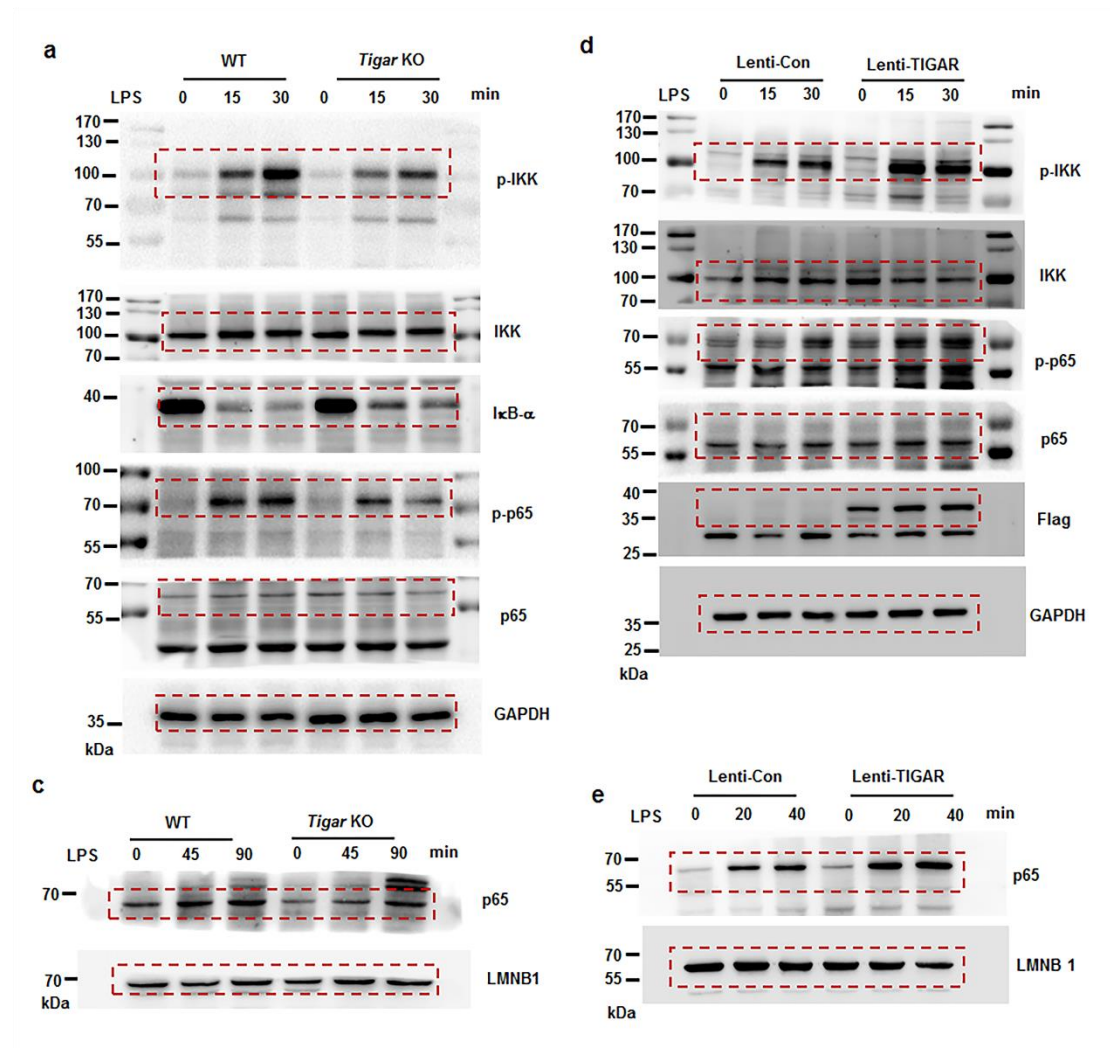

**Fig 4**

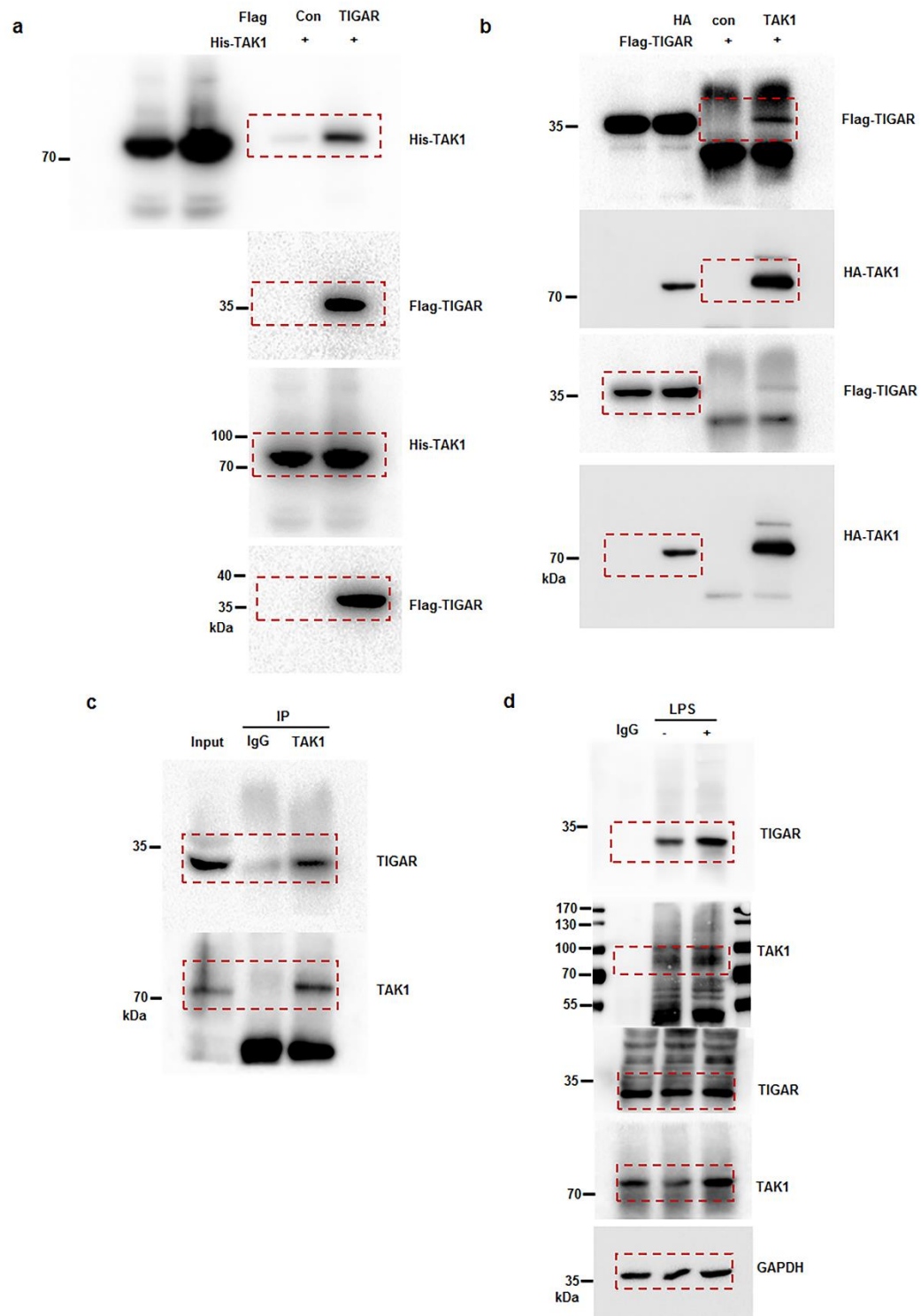

**Fig 4**

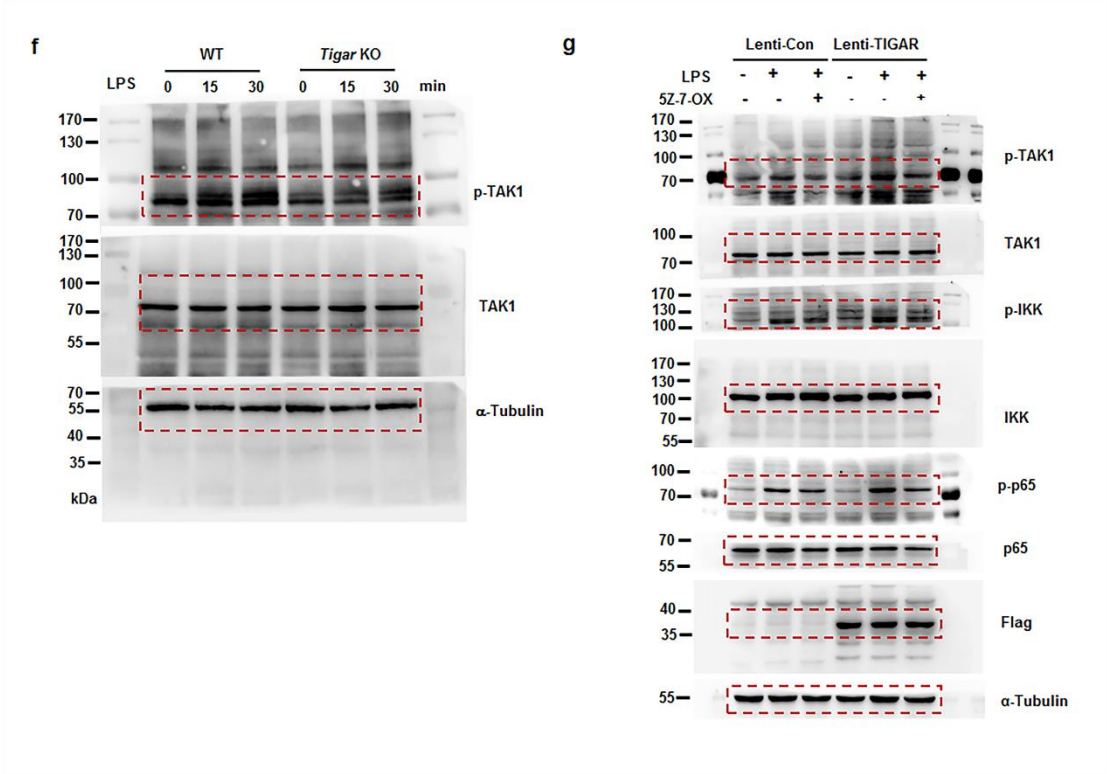

**Fig 5**

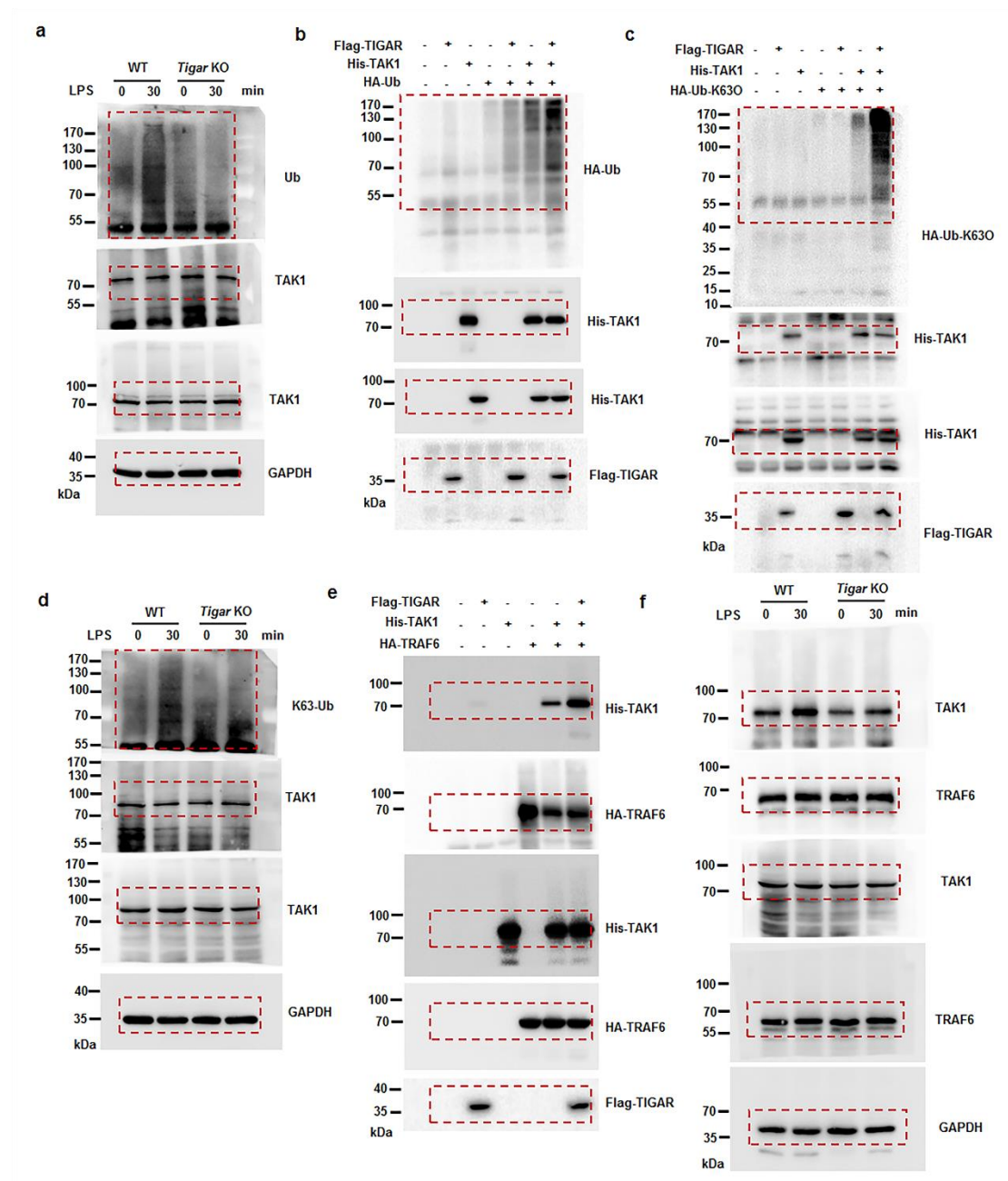

**Fig 5**

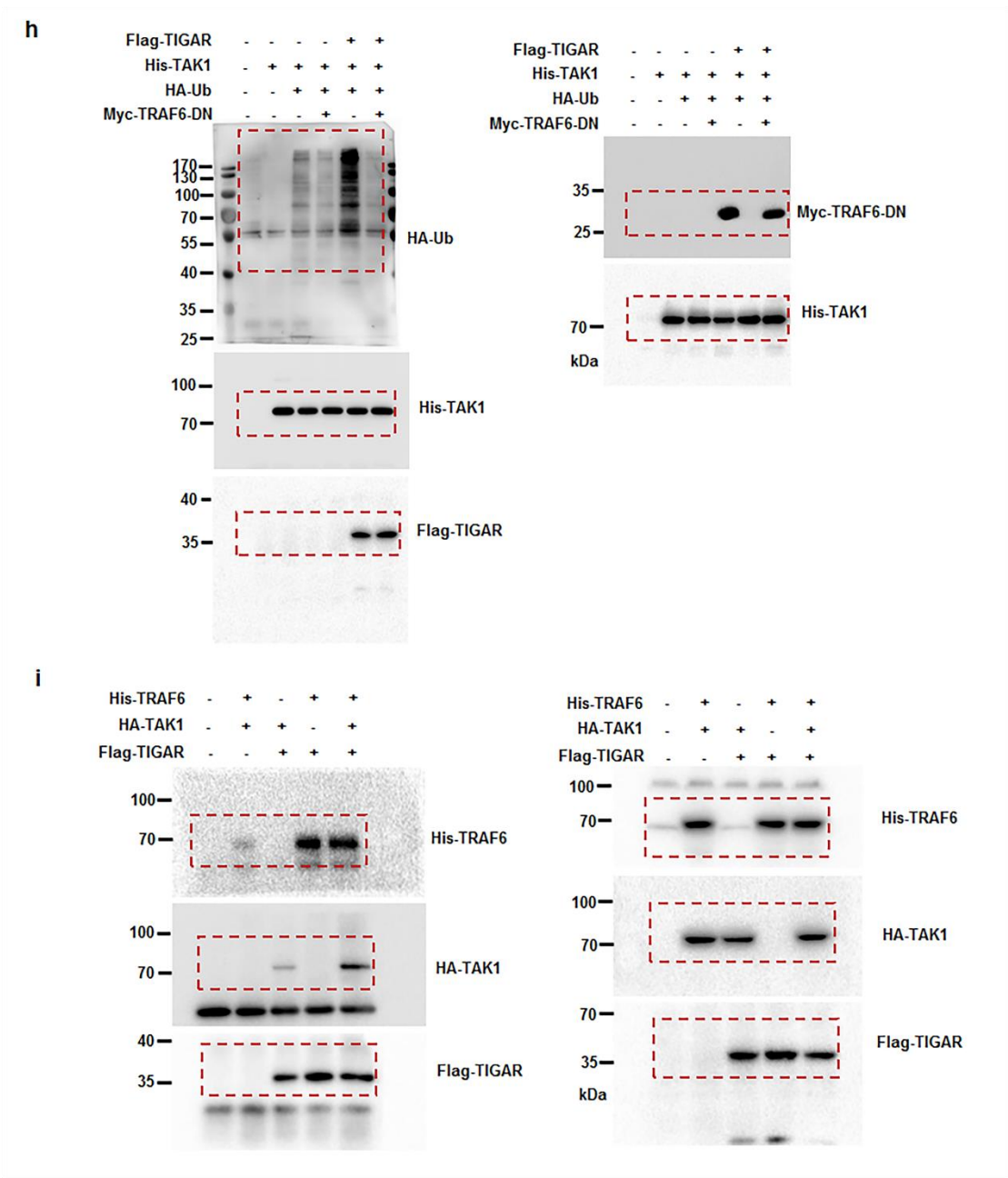

Fig 5

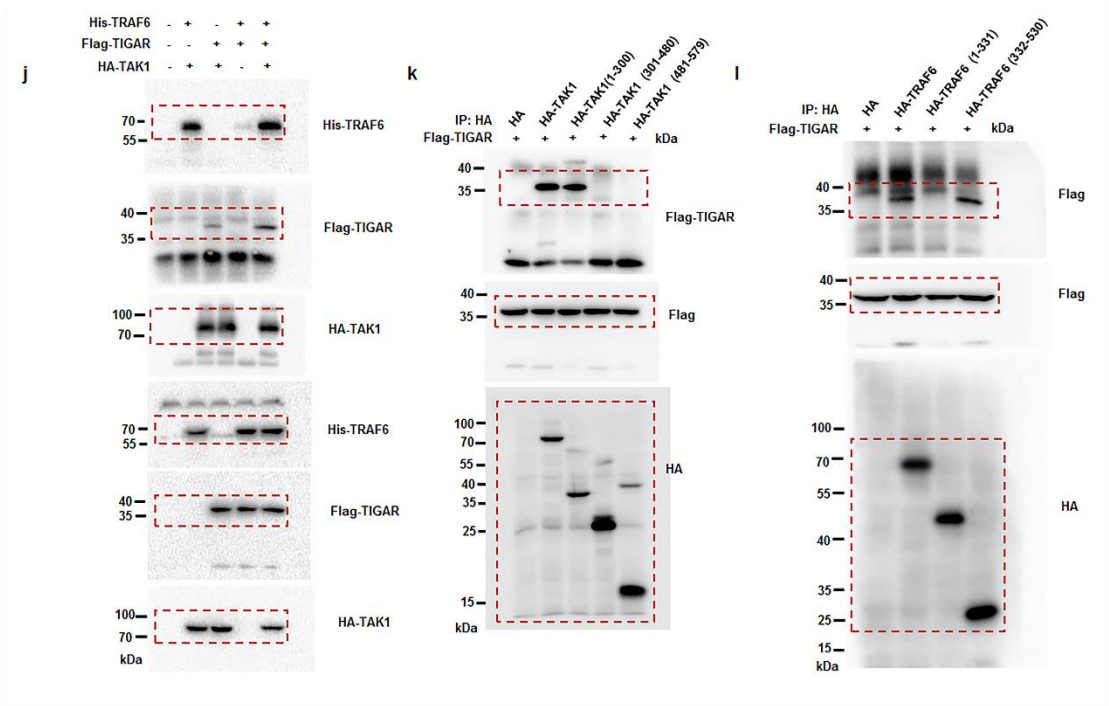

Fig 6

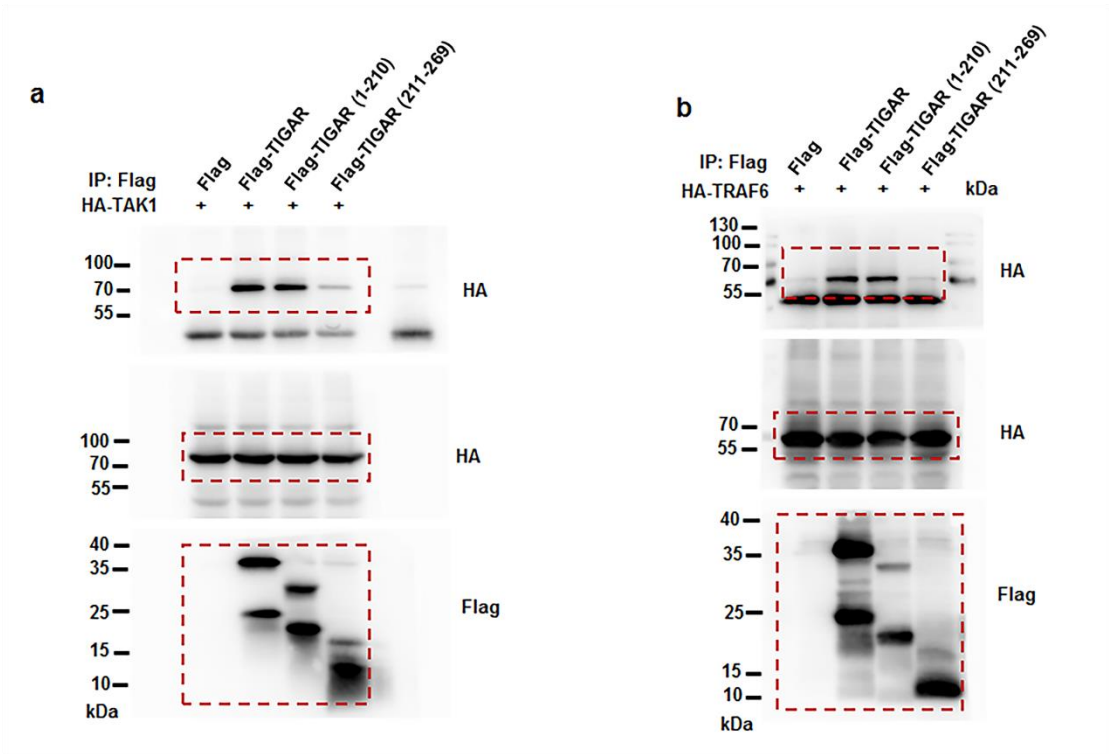

**Fig 6**

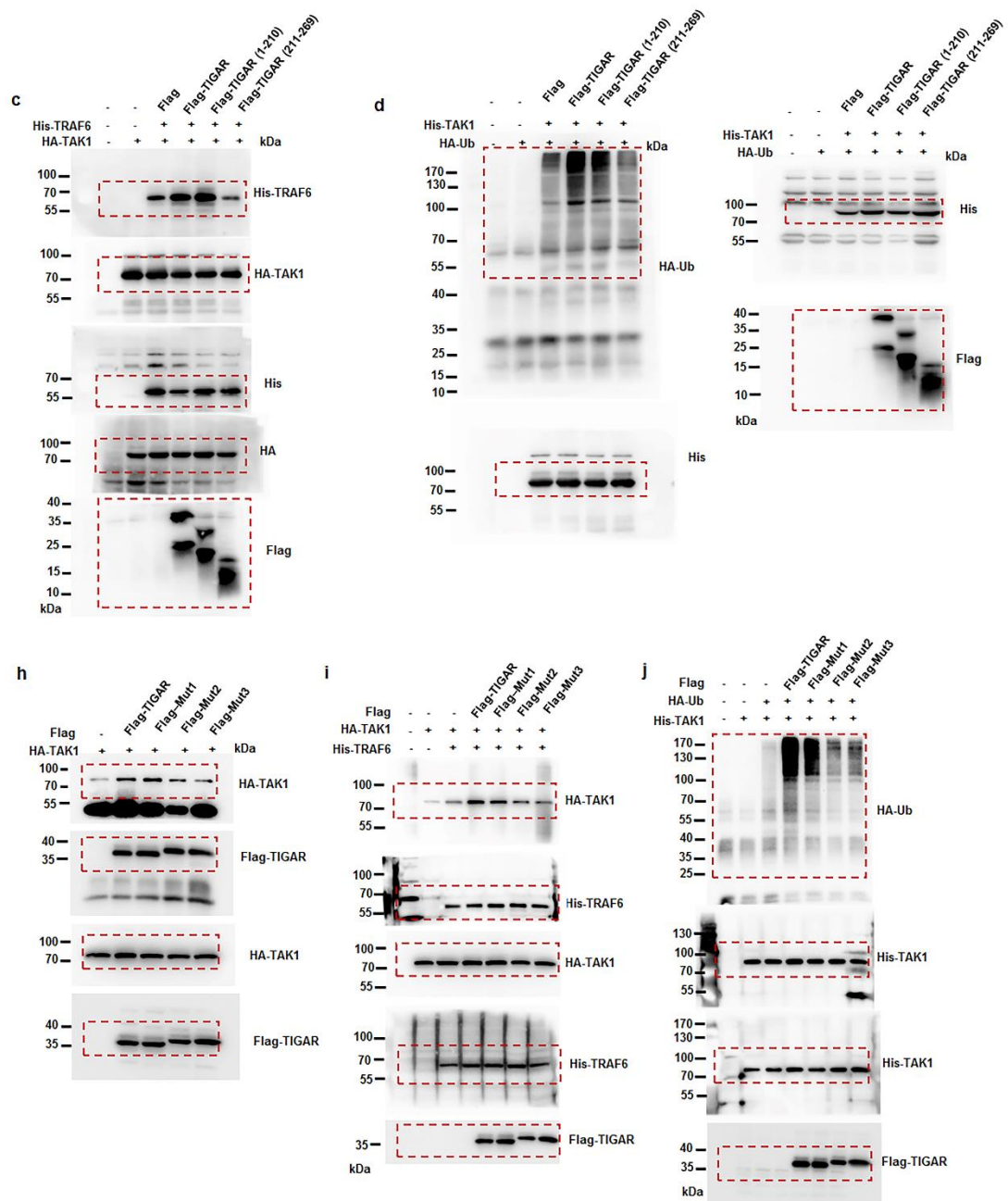

Fig 7

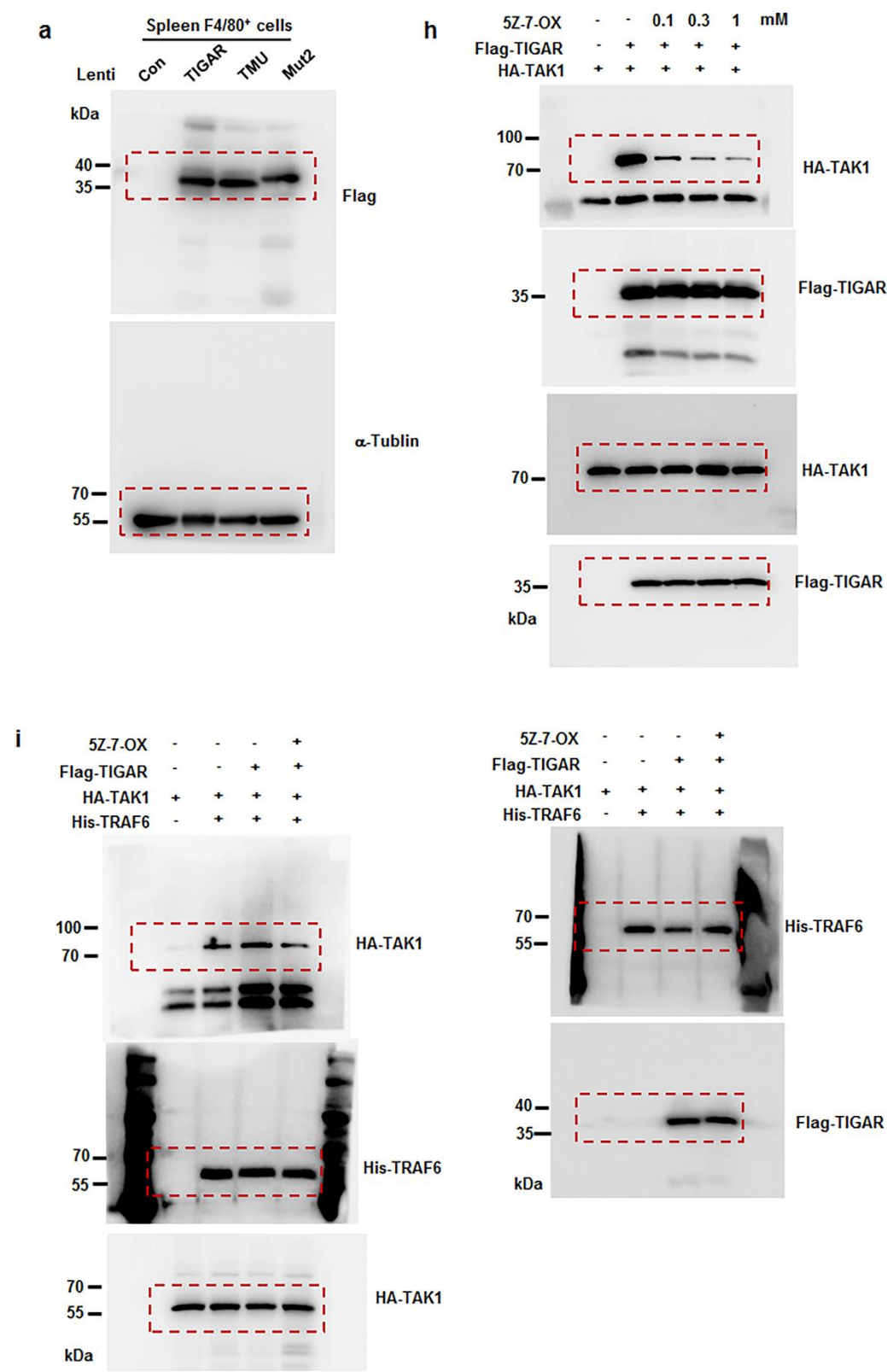

Supplement: Supplementary file 4 — Source Data [file 41467_2024_48708_MOESM4_ESM.zip › Source data/Uncropped scans of blots.pdf]
